# Integrated genetic and pharmacologic interrogation of rare cancers

**DOI:** 10.1038/ncomms11987

**Published:** 2016-06-22

**Authors:** Andrew L. Hong, Yuen-Yi Tseng, Glenn S. Cowley, Oliver Jonas, Jaime H. Cheah, Bryan D. Kynnap, Mihir B. Doshi, Coyin Oh, Stephanie C. Meyer, Alanna J. Church, Shubhroz Gill, Craig M. Bielski, Paula Keskula, Alma Imamovic, Sara Howell, Gregory V. Kryukov, Paul A. Clemons, Aviad Tsherniak, Francisca Vazquez, Brian D. Crompton, Alykhan F. Shamji, Carlos Rodriguez-Galindo, Katherine A. Janeway, Charles W. M. Roberts, Kimberly Stegmaier, Paul van Hummelen, Michael J. Cima, Robert S. Langer, Levi A. Garraway, Stuart L. Schreiber, David E. Root, William C. Hahn, Jesse S. Boehm

**Affiliations:** 1 Boston Children's Hospital, 300 Longwood Avenue, Boston, Massachusetts 02115, USA; 2 Dana-Farber Cancer Institute, 450 Brookline Avenue, Boston, Massachusetts 02215, USA; 3 Broad Institute of Harvard and MIT, 415 Main Street, Cambridge, Massachusetts 02142, USA; 4 Koch Institute for Integrative Cancer Research at MIT, 500 Main Street, Cambridge, Massachusetts 02139, USA; 5 Brigham and Women's Hospital, 75 Francis Street, Boston, Massachusetts 02115, USA; 6 Howard Hughes Medical Institute, Chevy Chase, Maryland 20815, USA

## Abstract

Identifying therapeutic targets in rare cancers remains challenging due to the paucity of established models to perform preclinical studies. As a proof-of-concept, we developed a patient-derived cancer cell line, CLF-PED-015-T, from a paediatric patient with a rare undifferentiated sarcoma. Here, we confirm that this cell line recapitulates the histology and harbours the majority of the somatic genetic alterations found in a metastatic lesion isolated at first relapse. We then perform pooled CRISPR-Cas9 and RNAi loss-of-function screens and a small-molecule screen focused on druggable cancer targets. Integrating these three complementary and orthogonal methods, we identify CDK4 and XPO1 as potential therapeutic targets in this cancer, which has no known alterations in these genes. These observations establish an approach that integrates new patient-derived models, functional genomics and chemical screens to facilitate the discovery of targets in rare cancers.

## Abstract (web summary)

Identifying therapeutic targets in rare cancers is challenging due to the lack of relevant pre-clinical models. Here, the authors generate a cancer cell line from a paediatric patient with a rare undifferentiated sarcoma and through functional genomics and chemical screens identified CDK4 and XPO1 as potential therapeutic targets in this cancer.

## Graphical abstract

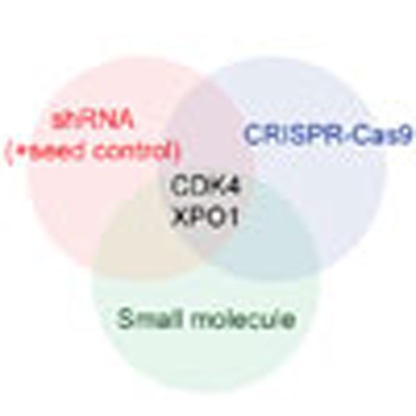

Despite large-scale efforts to identify genetic alterations that predict sensitivity to specific small molecules, the type and number of established cancer cell lines do not yet represent the full spectrum of human cancers^1^,^2^,^3^. In particular, the lack of patient-derived models has slowed the identification of targets and the development of new therapeutic agents for paediatric and other rare solid tumour cancers^4^. For rare paediatric cancers, the lack of preclinical data has required clinicians to rely on case reports, clinical intuition or empiricism to create treatments for such cancers.

Recent advances in methods to propagate patient-derived cell lines provide an opportunity to obtain representative cell lines from such rare cancers^5^,^6^,^7^. Furthermore, massively parallel-sequencing technology permits one to profile these cancers to ensure that the cell line recapitulates the genetic alterations found in the tumour tissue. We hypothesized that the systematic assessment of dependencies using CRISPR-Cas9, RNA interference (RNAi) and small-molecule profiling approaches in early passage patient-derived models from rare cancers would facilitate the identification of potential cancer dependencies. We define a dependency as a gene that when suppressed with short hairpin RNA (shRNA), deleted with CRISPR-Cas9 or inhibited with a small molecule leads to decreased proliferation or survival.

Here, we have derived a patient-derived cancer cell line, CLF-PED-015-T, from a paediatric patient with a rare undifferentiated sarcoma. We show the feasibility of performing pooled CRISPR-Cas9 loss-of-function, RNAi dependency and small-molecule screens in parallel. When we integrate these complementary and orthogonal methods, we identify and evaluate CDK4 and XPO1 as potential therapeutic targets in this cancer. These observations provide evidence that combining new patient-derived models, functional genomics and chemical screens facilitates the discovery of targets in rare cancers.

## Results

### Derivation and characterization of CLF-PED-015-T

**Figure 1:**
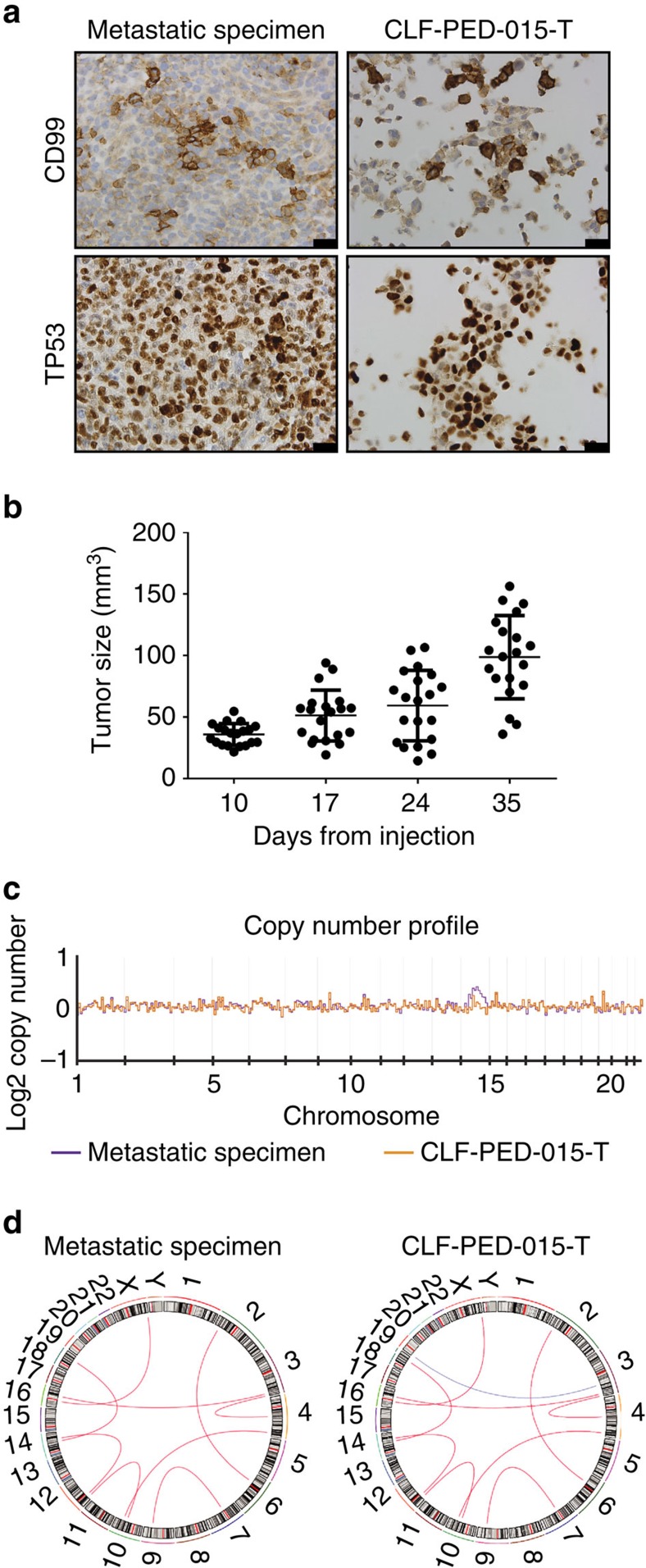
Patient-derived CLF-PED-015-T cell line recapitulates features of the metastatic tissue. (**a**) Immunohistochemistry of CD99 and p53 performed on the metastatic tissue and CLF-PED-015-T. Images taken at 60 × magnification. Scale bars, 10 μm. (**b**) Subcutaneous tumour volume of CLF-PED-015-T (*n*=20). Error bars represent mean±s.d. (**c**) Estimated log2 number of copies/ploidy comparing tumour and cell line showed no significant differences. (**d**) Circos plot identifying fusion events (see Methods section). Nine fusions were identified in the metastatic sample after first relapse by RNA-sequencing. These fusions were found in the CLF-PED-015-T cell line either by RNA-sequencing or quantitative reverse transcription PCR (red line). One additional fusion was observed in the cell line (blue line).

As a proof-of-concept, we derived a cell line, CLF-PED-015-T, from a paediatric patient with a multiply relapsed rare metastatic undifferentiated sarcoma (Supplementary Fig. 1a). We obtained metastatic tissue after the patient's first relapse and performed whole-exome sequencing (WES) and RNA-sequencing. The patient then received radiation and chemotherapy (temozolomide and irinotecan), but ultimately relapsed and a second biopsy was obtained. We generated a cell line from this second biopsy (CLF-PED-015-T), which exhibited similar histomorphology and immunohistochemical features (for example, CD99+ and TP53+) to the metastatic tissue obtained at first relapse (see Methods section; Fig. 1a). We also found that CLF-PED-015-T formed tumours when injected subcutaneously in immunodeficient mice (Fig. 1b) at rates similar to that observed with the well-established neuroblastoma BE(2)C and Ewing sarcoma TC-32 cell lines (Supplementary Fig. 1b). We then performed WES and RNA-sequencing on CLF-PED-015-T. When we compared the metastatic tissue from first relapse with CLF-PED-015-T, the copy number profiles were similar (Fig. 1c). As expected, this paediatric sarcoma harboured relatively few somatic nucleotide substitutions, although we note the existence of additional point mutations in the cell line as compared with the metastatic tissue from primary relapse (Supplementary Table 1). In the tissue from the metastatic lesion at first relapse, we identified nine fusion events detected by three RNA-sequencing fusion discovery algorithms including *RPS24-EMP1*, *TMED10-ITPR2*, *PRKCH-ALPK2*, *FUS-GABRA3*, *IGF2BP2-AMFR*, *UBXN7-GRID2*, *GALNT7-PDLIM1*, *VEGFA-DTNB*, and *ZNF680-XPA* (see Methods section; Fig. 1d; Supplementary Table 2). We detected these same fusions either by RNA-sequencing or by quantitative reverse transcription PCR in CLF-PED-015-T (Fig. 1d; Supplementary Table 2; Supplementary Fig. 1c). In addition, we found a *FSD1-CLSTN2* fusion in CLF-PED-015-T. The observed differences between the first metastatic sample and the samples from which CLF-PED-015-T was generated are likely related to the radiation and chemotherapy treatments administered between the acquisition of these two samples. Collectively, these observations suggest that the CLF-PED-015-T cell line retains the major somatic alterations, histologic and tumorigenic properties of this rare undifferentiated sarcoma.

Although this cell line recapitulated many of the somatic alterations found in the metastatic tumour after the first relapse, we failed to identify somatic point mutations that suggested a therapeutic approach in either tissue or cell line using Precision Heuristics for Interpreting the Alteration Landscape (PHIAL)^8^, (Supplementary Table 1). Furthermore, none of the gene fusions involved genes encoding proteins targeted by existing, approved small molecule inhibitors (for example, BCR-ABL or EML4-ALK).

### Genetic and pharmacologic screens to identify targets

**Figure 2:**
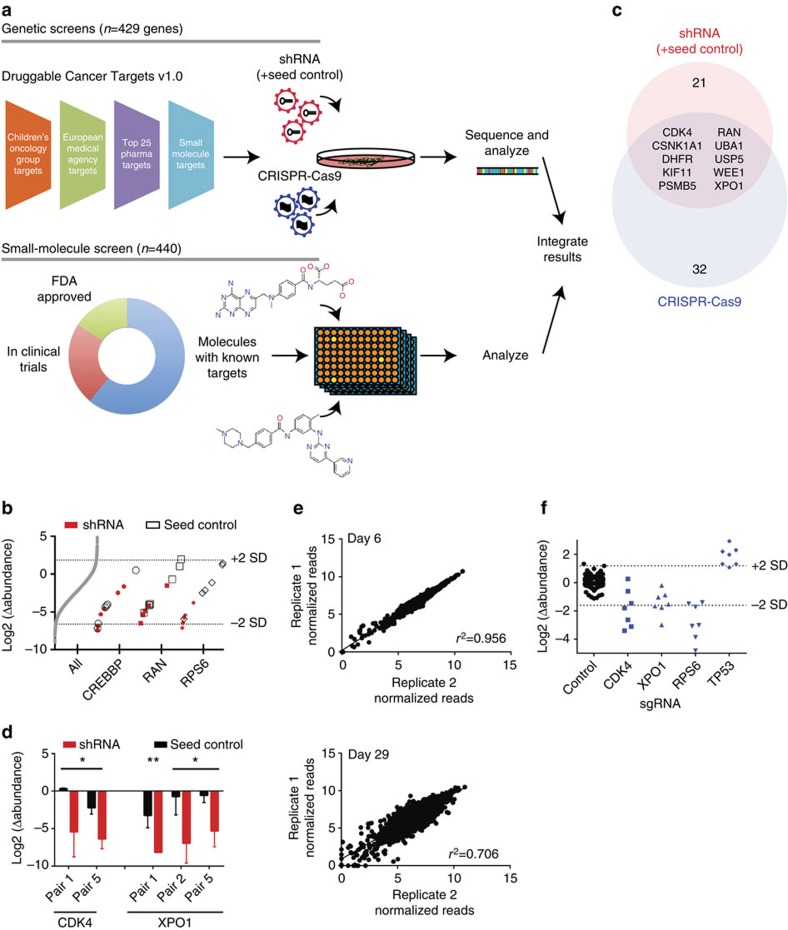
High-throughput functional genomic screens are feasible in an early passaged patient-derived model. (**a**) Schema for screens. shRNA and sgRNA libraries were created by compiling targets from the indicated sources and created the Druggable Cancer Targets v1.0 shRNA and sgRNA libraries. In parallel, a compound screen was performed utilizing 440 compounds identified previously^9^. (**b**) Using shRNA seed controls to identify off-target shRNAs. Distribution of shRNAs shown in grey. CREBBP was identified as a false positive due to the significant miRNA seed effects (comparing circle outlines, seed controls, to red dots, shRNAs). *RAN* alternatively was identified as a candidate when accounting for seed effects (square outlines, seed controls, to red squares). A positive control target, RPS6 showed a clear separation between seed controls (diamond outlines) and shRNAs (red diamonds). Each point represents the mean of four biological replicates. (**c**) Summary of RNAi and CRISPR-Cas9 screens. When comparing candidates from both screens, we found 10 genes that scored in both screens. (**d**) Detailed comparison of paired shRNAs with shRNA seed controls identified CDK4 and XPO1 in CLF-PED-015-T. Error bars represent mean±s.d. for four independent experiments. (**e**) Replicates are highly correlated in CLF-PED-015-T following introduction of sgRNAs at days 6 and 29. (**f**) CRISPR-Cas9 screens in CLF-PED-015-T. Each dot represents the mean of three replicates for a given sgRNA.

To identify potential therapeutic targets in CLF-PED-015-T, we performed both genetic (CRISPR-Cas9 and RNAi) and pharmacologic screens to identify genes or small molecules that decreased proliferation or survival and focused our attention on targets that were identified by all approaches. Specifically, we compiled a list of potentially druggable targets by combining the lists of targets of (i) biologically active compounds identified from large-scale cell line profiling experiments with established cancer cell lines^1^,^9^,^10^, (ii) small molecules in development from 25 pharmaceutical companies and (iii) small molecules that are the subject of Children's Oncology Group clinical trials or in the process of being approved by the European Medical Agency as of May 2014. In total, we identified a unique set of 429 druggable targets (Supplementary Table 3; Fig. 2a).

Although RNAi and CRISPR-Cas9 technologies both enable loss-of-function screens, RNAi induces gene suppression while CRISPR-Cas9-based genome editing results in gene deletion. As such these technologies have different off-target effects and mechanisms of gene suppression, and we used both approaches for the genetic screens to facilitate the identification of robust candidates. We created custom shRNA and CRISPR-Cas9 libraries targeting these 429 genes (see Methods section) along with six genes identified as essential genes based on our prior genome-scale shRNA screens (for example, *RPS6*, *RPL7*, *SFPQ*)^11^. These genes, when suppressed with RNAi, induce a significant decrease in proliferation/viability across the majority of cell lines tested. In addition to 262 shRNA controls that target genes not present in the human genome (for example, green fluorescence protein (GFP), luciferase), we included controls that identify off-target effects from miRNA-based seed sequences (referred to as seed controls) to minimalize the identification of false positives. Specifically, for each shRNA (*n*=3,056), we included a seed control shRNA that disrupts RNAi activity against the intended target for each experimental shRNA, yet retains the 6–8 nucleotide miRNA-based seed sequence^12^,^13^. For the CRISPR-Cas9 single guide (sgRNA) library, there were an additional 375 sgRNA controls that target genes not present in the human genome. In total, the shRNA library contained 6,374 shRNAs and the CRISPR-Cas9 library contained 3,372 sgRNAs. These shRNAs and sgRNAs were introduced into lentiviral vectors to create two Druggable Cancer Targets (DCT v1.0) libraries (see Methods section; Fig. 2a).

### CDK4 and XPO1 as potential therapeutic targets

For the shRNA DCT v1.0 screen, we optimized and introduced this library into CLF-PED-015-T and A549, an established lung cancer cell line, in a pooled format and evaluated the abundance of the shRNAs after 26 days using massively parallel-sequencing (see Methods section; Supplementary Fig. 2a,b). We first performed analyses that focused on matching the experimental and seed control shRNAs to confirm the reproducibility and on-target specificity for individual constructs. For strong hits such as known essential genes (for example, *RPS6*), we noted significant differences between seed control and paired shRNAs (Fig. 2b; Supplementary Fig. 3a). For some genes, we failed to find a difference between seed control and shRNA (Fig. 2b, *CREBBP*; Supplementary Fig. 3b, *XIAP1*), indicating that the proliferation effects were likely attributable to seed-mediated effects rather than the intended target gene. Using the differential abundance between the seed control and shRNA, we collapsed the individual shRNA effects to consensus gene dependencies by subjecting these paired shRNAs to RNAi Gene Enrichment Ranking^14^. We found that all the six control essential genes were essential in CLF-PED-015-T (*P* values by the student's *t*-test <0.028). In addition, we found that 21 of 429 genes (5%) scored in CLF-PED-015-T with *P* values <0.05 (Fig. 2c; Supplementary Table 4), which was similar to the number of genes that scored in A549 (Supplementary Table 5).

Among the 21 candidates that scored with *P* values <0.05 in CLF-PED-015-T, we found that these candidates included members of the proteasomal pathway (for example, *UBA1*, *PSMD1*, *PSMD2* and *PSMB5*), the DNA damage response/repair pathway (for example, *WRN*, *POLA1*, *RAD51* and *WEE1*), genes involved in nuclear export (for example, *RAN* and *XPO1*) and cyclin dependent kinases (for example, *CDK1* and *CDK4*). The differences between shRNA seed control and paired shRNA for CDK4 and XPO1 are shown in Fig. 2d.

When we examined the CRISPR-Cas9 screens, we compared the representation of sgRNAs at 6 days to that at 29 days to identify genes enriched or depleted in this screen. We achieved an average representation rate of a minimum of 1,000 cells per sgRNA for both cell lines per replicate (Supplementary Fig. 4a). We observed a similar distribution of sgRNAs in both CLF-PED-015-T and A549, demonstrating that there was no clear technical difference in screening this patient-derived cell line and an established cancer cell line (Supplementary Fig. 4b,c). Furthermore, we found that biological replicates were highly correlated at the final time point (Fig. 2e; Supplementary Fig. 4d). In addition, the range of differential abundance for CLF-PED-015-T between controls and known essential genes identified from RNAi screens (for example, *RPS6*) was similar to that observed in A549s (Fig. 2f; Supplementary Fig. 4e).

Using RNAi Gene Enrichment Ranking^14^, we collapsed the sgRNAs in a similar fashion to the shRNAs, but we set a threshold that four of seven sgRNAs must rank in the top 500 candidates to be considered further. When we analyzed the data from the CRISPR-Cas9 screen, four of six essential genes identified from prior RNAi screens (*RPS6*, *RPL7*, *EIF3G* and *SFPQ*) exhibited *P* values by the student's *t*-test <0.041. We then identified 32 genes that showed *P* values <0.05 (Fig. 2c; Supplementary Table 6). The top gene ranked was SP1 (*P* value=0.0002), a transcription factor, which when suppressed, can lead to tumour suppression in mesothelioma xenografts^15^. Three components of the nuclear export receptor complex were identified (for example, *XPO1*, *KPNB1* and *RAN*) along with the cyclin dependent kinase, *CDK4*. In addition, we found *TOP2A* and *DHFR*, which are targets of doxorubicin and methotrexate.

When we compared the results of the shRNA and CRISPR-Cas9 genetic screens, we found 10 of the 32 genes in the CRISPR-Cas9 screen scored in both the screens (*RAN*, *UBA1*, *KIF11*, *XPO1*, *USP5*, *CSNK1A1*, *PSMB5*, *CDK4*, *WEE1* and *DHFR*; Fig. 2c; Supplementary Table 7). This rate of concordance was similar to what we observed when we compared the genes that overlapped between RNAi and CRISPR-Cas9 screens in A549 cells (53% or 20 genes of 38 genes, Supplementary Tables 8 and 9). On the basis of our experience in screening hundreds of cancer cell lines^11^,^16^, the rate of concordance between screens that we observed with CLF-PED-015-T or A549 is typical due to the differences in how RNAi and CRISPR-Cas9 affect gene expression.

**Figure 3:**
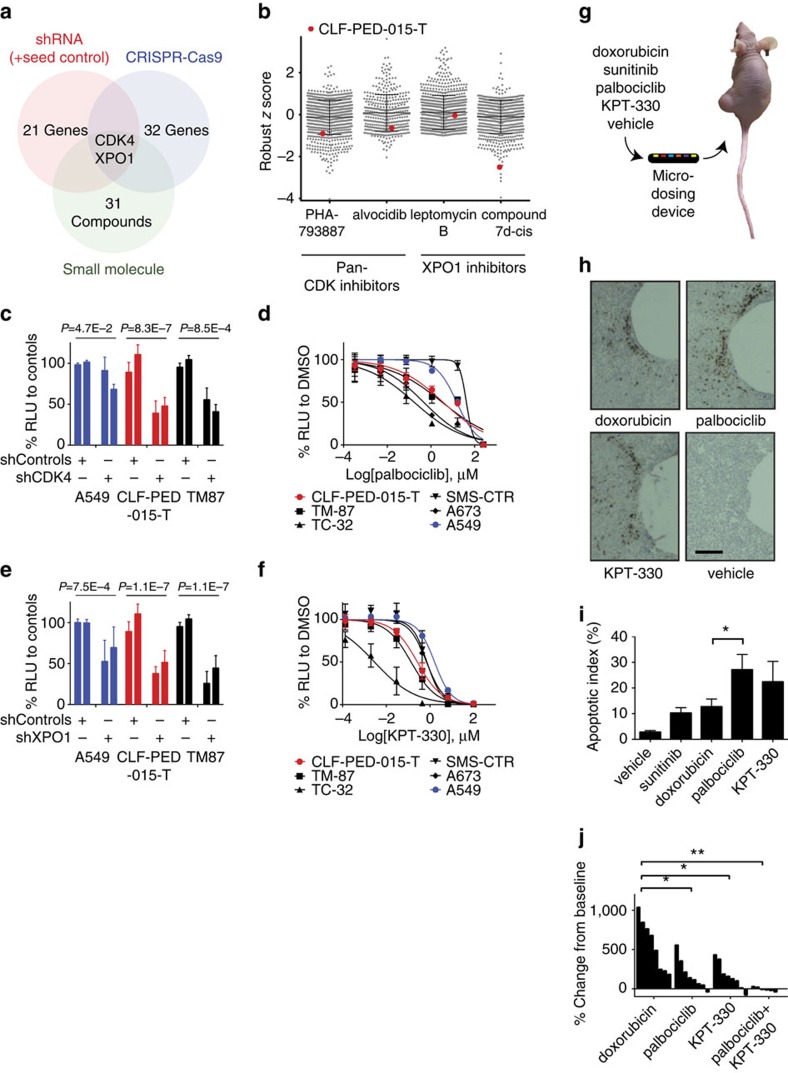
High-throughput genetic screens and compound screens identify CDK4 and XPO1 as potential targets. (**a**) Summary of RNAi, CRISPR-Cas9 and small-molecule screens. (**b**) Comparison of CLF-PED-015-T sensitivity of pan-CDK and XPO1 inhibitors with CCLE cell lines. Values are based on a robust *z* score. (**c**) shRNA (*n*=2) validation of dependency on CDK4. Error bars represent mean±s.d. for at least three independent experiments. (**d**) Effects of palbociclib on cell viability. Error bars represent mean±s.d. for four independent experiments. (**e**) shRNA (*n*=2) targeting XPO1. Error bars represent mean±s.d. for at least three independent experiments. (**f**) Effects of KPT-330 on cell viability. Error bars represent mean±s.d. for three independent experiments. (**g**) Schema depicts implantable microdevice used for *in vivo* assessment of drugs. (**h**) Sample images of tumour regions in which drugs released from the microdevices cause apoptosis as measured by cleaved caspase-3 expression (brown cells) for the indicated agents. Scale bars, 200 μm. (**i**) Quantitative analysis of apoptotic index for each of the tested agents. Error bars represent mean±s.d. *P value by the student's *t*-test=0.05. (**j**) Waterfall plot indicating the tumour response following treatment for mice harbouring CLF-PED-015-T subcutaneous xenografts following 25 days of treatment. Once tumours grew to 100–200 mm^3^, mice were treated with doxorubicin, palbociclib, KPT-330 or the combination of palbociclib and KPT-330 (see Methods section) and monitored over 25 days. *P values by the student's *t*-test <0.05, **P<0.005.

In parallel to the functional genomic screens, we performed a small-molecule screen in CLF-PED-015-T using a library of small molecules with known targets^9^. This library of 440 compounds includes 268 probes, 100 drugs in clinical trials and 72 FDA approved drugs (Fig. 2a; Supplementary Table 10). We tested each compound in 8-point concentration ranges in duplicate, and calculated an area under the curve (AUC) for each compound (defined by prior studies^1^,^9^,^10^). We considered an AUC <0.5 as significant for decreasing proliferation based on prior studies (see Methods section). Of 407 evaluable compounds, 31 (8%) compounds significantly decreased cell viability (Supplementary Table 11) including pan-CDK inhibitors (dinaciclib, alvocidib, SNS-032), topoisomerase inhibitors (SN-38, topotecan, doxorubicin), inhibitors of nicotinamide phosphoribosyltransferase (NMPRTase) (daporinad, CAY10618 and GMX-1778) and leptomycin B, an inhibitor of nuclear export. Integrating the small-molecule screen with the genomic screens, we found that depleting CDK4 and XPO1 or targeting them with small molecules led to reduced cell viability (Fig. 3a).

### Validating CDK4 and XPO1 *in vitro* and *in vivo*

We first compared these results to previous results of treating 835 cell lines from the Cancer Cell Line Encyclopedia (CCLE) with pan-CDK or XPO1 inhibitors^9^,^10^. Robust *z* scores were calculated by using the median and absolute deviation from the median to remove bias from outlier data points. We found that CLF-PED-015-T scored in the top 20% of cell lines sensitive to PHA-793887 and alvocidib, both pan-CDK inhibitors, with robust *z* scores of −0.91 and −0.66, respectively (Fig. 3b). In addition, CLF-PED-015-T was ranked fifth among the CCLE cell lines with respect to the observed sensitivity to compound 7*d*-*cis*, a XPO1 inhibitor (robust *z* score of −2.51) (Fig. 3b).

Because previous pharmacologic profiling efforts have focused mostly on adult epithelial cancers^1^, we identified several comparable paediatric cancer cell lines including SMS-CTR (rhabdomyosarcoma), TC-32 (Ewing sarcoma), A673 (Ewing sarcoma) and TM-87 (extrarenal rhabdoid tumour) to compare to CLF-PED-015-T. To validate dependency of CLF-PED-015-T on CDK4, we first assessed viability of cell lines following depletion of CDK4 with shRNAs. Introduction of *CDK4*-specific shRNAs into CLF-PED-015-T and TM-87 induced more than a 50% decrease in proliferation when compared to shRFP and shLuc controls (Fig. 3c; Supplementary Fig. 5a). Similar to CLF-PED-015-T, we found that the CDK4/6 inhibitor, palbociclib, inhibited the proliferation of TC-32, A673 and TM-87 at low micromolar concentrations (Fig. 3d) when compared to SMS-CTR and A549. Recent work has implicated sensitivity to the CDK4/Cyclin D1 pathway inhibition in Ewing sarcomas and rhabdoid tumours^17^,^18^. We observed that treatment of CLF-PED-015-T with flavopiridol, a pan-CDK inhibitor or palbociclib or LEE011, CDK4/6 specific inhibitors, led to decreased phospho-Rb^Ser807/811^ and G0/G1 arrest but did not activate cleaved caspase-3 *in vitro* (Supplementary Fig. 5b–d).

We subsequently validated the consequences of XPO1 depletion or inhibition on viability in these cell lines. XPO1 depletion by shRNAs led to an ∼50% decrease in proliferation when compared with controls (Fig. 3e; Supplementary Fig. 5a). We also found that XPO1 inhibition with KPT-330, a compound in clinical trials, potently reduced cell viability at nanomolar concentrations (Fig. 3f). XPO1 is a member of the nuclear export complex and is known to regulate tumour suppressors, such as p53 and RB^19^. We observed that deletion of *TP53* provided CLF-PED-015-T a growth advantage in the pooled CRISPR-Cas9 screen (Fig. 2f) and that CLF-PED-015-T harbours wild-type *TP53.* Furthermore, *MDM2* scored in the CRISPR-Cas9 screen and *USP5* scored in both RNAi and CRISPR-Cas9 screens. Both genes are involved in mediating p53 ubiquitination^20^. On the basis of this, we assessed whether XPO1 depletion affected p53 levels and function. Treatment with KPT-330 led to nuclear accumulation of p53, activation of p21 and increased cleaved caspase-3 in CLF-PED-015-T (Supplementary Fig. 5c,e). We observed similar increases in p53 and p21 in TM-87 and TC-32, which also harbour wild-type *TP53*. We then tested whether p53 expression is necessary for the effects of inhibiting XPO1. We introduced sgRNAs specific for GFP and *TP53* into CLF-PED-015-T and the lung cancer cell line A549. After confirming that p53 expression was suppressed (Supplementary Fig. 6a), we failed to find a substantial difference in the response to KPT-330 (Supplementary Fig. 6b,c). These observations suggest that p53 is not the sole mediator of the response to inhibiting XPO1 in this setting, and that inhibiting or suppressing XPO1 decreased the proliferation of CLF-PED-015-T.

To confirm whether these findings extended to tumours growing *in vivo*, we tested the CDK4 and XPO1 dependencies using a micro-dosing device^21^. This device simulates systemic administration of compounds by releasing microdoses of drugs into confined regions of tumours at appropriate doses and allows for assessment of drug efficacy within the native tumour microenvironment. In previous work, this micro-dosing device recapitulated the effects of intravenous delivery of common chemotherapies, such as doxorubicin, paclitaxel, cisplatin and gemcitabine, to a patient-derived triple negative breast cancer tumour model^21^. Here, we delivered compounds targeting CDK4 and XPO1 into subcutaneous xenografts generated with the CLF-PED-015-T cell line using the micro-dosing device (Fig. 3g). For comparison, we included two control compounds, sunitinib and doxorubicin. Sunitinib exhibited an AUC of 0.71 and known targets of sunitinib (FLT1, KDR, FLT3, FLT4, PDGFRB, KIT, RET, CSF1R) did not score in either the RNAi or CRISPR-Cas9 screens. Doxorubicin exhibited an AUC of 0.42 and its target, *TOP2A*, ranked fourth in the CRISPR-Cas9 screen. We then compared single agent effectiveness with CDK4 or XPO1 inhibition to these compounds. We noted that the apoptotic indexes (% of cleaved caspase-3 positive cells versus total cells) for doxorubicin was 12.7% (±3%; *n*=4) and sunitinib was 10.3% (±2.1%; *n*=2), while palbociclib, a CDK4 inhibitor, induced an apoptotic index of 27.1% (±5.5%; *n*=4), and KPT-330, an XPO1 inhibitor, exhibited an apoptotic index of 22.4% (±7.6; *n*=4) after a 24-h exposure to the compound (Fig. 3h,i). These observations suggest that inhibiting CDK4 or XPO1 inhibits tumour growth *in vivo*.

To confirm these findings, we treated tumour xenografts with doxorubicin, palbociclib and KPT-330 as single agents, and palbociclib and KPT-330 in combination for 25 days. Doxorubicin treatment failed to substantially inhibit subcutaneous tumour growth, confirming anecdotal clinical experiences with this drug (Fig. 3j). In contrast, treatment with KPT-330 or palbociclib induced significant tumour stabilization (Fig. 3j). Furthermore, we found that the combination of palbociclib and KPT-330 was much more efficacious than either agent alone (Fig. 3j).

## Discussion

Rare cancers account for up to 25% of adult cancers and 10% of paediatric cancers^22^,^23^. Clinical outcomes for patients with rare cancers are generally inferior to those with more common cancers in large part due to the absence of sufficient clinical or preclinical data to guide decision-making. As a result, patients and physicians often rely on anecdotal clinical evidence to select therapy^23^.

The lack of patient-derived models in rare cancers has slowed efforts to generate sufficient preclinical data to identify targets^24^. When the cell models exist (for example, H2228 cell line representing *EML4-ALK* rearranged non-small cell lung cancer (NSCLC), which occurs in ∼5% of NSCLC), preclinical studies using such cell lines have helped to identify therapeutic targets and accelerated clinical testing^25^,^26^. We generated CLF-PED-015-T as part of a systematic effort to develop cell lines from rare paediatric cancers. CLF-PED-015-T recapitulates many attributes of this patient's tumour before additional radiation and chemotherapy and thus provides an important reagent that facilitated genetic and pharmacologic studies. Future studies that include the analysis of tumours and the generation of cell lines at different points during the course of a patient's disease will facilitate our understanding both how the cancer evolves *in situ* in response to therapy as well as how closely cell lines generated from such tumours reflect any changes that occur.

Although both RNAi and CRISPR-Cas9 have been used in established human cells to perform loss-of-function studies, the use of these two technologies in a patient-derived cell line allowed us to mitigate the off-target effects of both approaches. Specifically, these two technologies differ in the mechanisms by which they affect gene expression. Specifically, RNAi induces gene suppression and is subject to miRNA-like off-target effects. Here, we used controls for each shRNA that eliminate the on-target effects of each shRNA allowing us to identify those shRNAs that exhibit largely on-target effects. In contrast, genome editing by CRISPR-Cas9 induces DNA breaks that are inaccurately repaired leading to frameshift mutations and small deletions^27^ and in most cases, effectively results in gene deletion. Moreover, the use of both approaches to nominate a candidate mitigates off-target effects and provides important information regarding the robustness of such findings, which is particularly important when analyzing a small number of cell lines.

The generation of a curated small-molecule collections enable high-throughput assessment of compounds, which can inhibit individual or multiple targets^10^. For individual cell lines, identifying true positives is difficult. Coupling the small molecule screen with genetic screens, we focused on *CDK4* and *XPO1*, which ranked in each of these approaches. CDK4/6 inhibitors were approved for advanced breast cancers in 2015 and have been implicated as potential therapeutic targets in Ewing sarcoma, a rare paediatric bone tumour^17^,^28^. XPO1 inhibition has induced objective responses in solid tumour xenografts of the Paediatric Preclinical Testing Programme^29^. The identification of *CDK4* and *XPO1* provide evidence to support testing of these agents in other paediatric undifferentiated sarcomas.

Taken together, these observations demonstrate that functional genomic and pharmacologic profiling of early passage patient-derived rare cancer preclinical models is feasible. These reveal *CDK4* and *XPO1* as potential targets for a paediatric undifferentiated sarcoma that does not have genomic alterations or amplifications in these genes. Although prior efforts to use specific technologies applied to patient-derived samples have not always been predictive^30^,^31^,^32^,^33^, the strategy described here differs from prior efforts by confirming that the genomic and phenotypic profile of the cell model recapitulates the metastatic tumour and by focusing on targets identified by the intersection of RNAi, CRISPR-Cas9 and pharmacologic approaches. The expansion of these types of studies may facilitate the identification of potential therapeutic targets for patients with rare cancers.

## Methods

### Derivation of CLF-PED-015-T

Tissue was used to create cell line models under IRB approved Dana-Farber Cancer Institute protocols. De-identified samples were obtained within 4 h following the resection of a metastatic brain lesion. The sample was minced into 1–2 mm^3^ cubes and digested with Collagenase IV (ThermoFisher Scientific, Waltham, MA, USA) for ∼3 h. Cells were seeded in six-well plates with RPMI and 10% fetal bovine serum. Cells were serially passaged after reaching 80% confluence.

### Whole-exome sequencing (WES)

WES was performed from cell pellets, matched-tumour tissue and germline DNA extracted from blood at the Broad Institute Genomics Platform using the Illumina HiSeq 2000 platform. The Agilent SureSelect Human All Exon v2 kit was used for capture and paired end 76 bp reads were obtained. This resulted in a mean target coverage of 102 × for the tumour and 57 × for CLF-PED-015-T. We filtered out common single nucleotide polymorphism (SNPs) that were validated in dbSNP and likely artifacts by setting minimum coverage and allele frequency thresholds.

### RNA sequencing

RNA-sequencing of the metastatic tissue was performed at the Broad Institute Genomics Platform using the Illumina TruSeq Platform. Library construction protocols for the CLF-PED-015-T were performed as previously reported^34^. Read alignment to reference genomes and fusion discovery was performed using the PRADA, Chimerascan and STAR algorithms^35^,^36^,^37^.

### Quantitative RT-PCR

Cells were grown and collected at log-phase growth. RNA was extracted using RNeasy Mini Kit (Qiagen, Germantown, MD, USA). RNA was normalized using Nanodrop 2000 (ThermoFisher Scientific) and cDNA libraries were created with High-Capacity cDNA Reverse Transcription Kit (ThermoFisher Scientific). cDNA libraries were amplified with Power SYBR Green on a QuantStudio6 with optimized primers for various fusions (Supplementary Table 12 for primers used in this study; ThermoFisher Scientific).

### Cell lines

A549 cells were obtained from ATCC (Manassas, VA, USA) and authenticated by Fluidigm fingerprinting. TM-87 and A673 were obtained from the Broad Institute Biological Samples Platform. TC32 and BE(2)C were verified by SNP analysis and provided by the Stegmaier Lab. CLF-PED-015-T passages and uses at different time points were authenticated by Fluidigm SNP analysis. Before DCT shRNA/CRISPR or small-molecule profiling, cells were tested for mycoplasma contamination.

### DCT v1.0 shRNA/CRISPR libraries and pooled screens

Gene-targeting shRNAs and appropriate seed controls^13^ were synthesized as single stranded DNA on an Agilent chip. shRNA sequences were selected using algorithms designed by the Broad Institute Genetic Perturbation Platform (GPP) (http://www.broadinstitute.org/rnai/public/). Oligonucleotides were designed with full hairpin sequences, flanked by restriction enzyme recognition sequences and PCR primer sites used to amplify double stranded DNA. The oligonucleotide pools were amplified using NEBNext kits (New England Biolabs, Ipswich, MA, USA). The PCR products were purified using Qiagen PCR cleanup kits and cloned into TRC005. The sgRNA pool was synthesized in a similar manner using the rule set described at the GPP portal^38^ and cloned into pXPR_BRD003 using Golden Gate cloning reactions. Both pooled libraries were amplified using electro-competent Stbl4 cells. Viruses from both pools were generated as outlined at the GPP portal. As CLF-PED-015-T was expanded, we performed titration of control viral constructs with lentiviral vectors to test if these cells could be transduced (Supplementary Fig. 2a,b).

For the CRISPR pool, CLF-PED-015-T was first transduced with the Cas9 expression vector pXPR_BRD111(Supplementary Fig. 2c)^39^. The DCT v1.0 libraries were subsequently transduced into CLF-PED-015-T in duplicate with two biological repeats (shRNA pool) and in cells harbouring the pXPR_BRD111 vector in triplicate (CRISPR pool) at an early passage (<30) and at an multiplicity of infection (MOI) <1, at a mean representation rate of 1,000 cells per sgRNA or shRNA. The shRNA-pooled virus library included 262 non-targeting controls and the CRISPR-pooled virus library included 375 non-targeting controls. Cells were passaged every 4–5 days until day 26 (shRNA pool) or day 29 (CRISPR pool) when cells were collected. Genomic DNA was extracted and was submitted for sequencing of the barcodes. We achieved sequencing depths of at least 500 reads per shRNA and 1,000 reads per sgRNA. For the shRNA DCT v1.0 screen, each seed control was matched and compared to the paired targeting shRNA. Overall, 119 pairs (5%) were removed due to poor representation in the virus pool. An average of 6.7 seed/shRNA pairs were available for analysis (Supplementary Fig. 2d).

### Small-molecule profiling

Cells were plated at 2,000 cells per well in a 384-well format, spun down briefly and incubated overnight at 37 °C/5% CO_2_. The next day compounds were pin transferred (CyBio Vario) into duplicate assay plates from 384-well plate stocks^9^,^10^ having 8-point, twofold concentration ranges. Assay plates were then incubated for 72 h and cell viability was measured using CellTiter-Glo (Promega, Madison, WI, USA). To calculate AUC, we averaged the primary sensitivity and normalized luminescence values to vehicle (DMSO) treatment and background (media-only) wells. 8- (CLF-PED-015-T) and 16- (data from the Cancer Therapeutics Response Portal http://www.broadinstitute.org/ctrp/) point concentration curves were computed and the AUC for each compound-cell line pair was calculated by integration under the response curve using Pipeline Pilot and MATLAB as previously described^9^. To render these values comparable with each other, each AUC was divided by (*n*−1), where *n* is the number of points in the concentration curve (that is, 7 for CLF; 15 for CTRP).

### CDK4/XPO1 target validation experiments

shRNAs were obtained from the Broad Institute Genomic Perturbation Platform (sequences in Supplementary Table 13; Broad Institute, Cambridge, MA, USA). shRNAs were sequenced and verified. After sequence verification, constructs were transfected with packaging vectors into HEK 293 T with TransIT-LT1 (Mirus Bio LLC, Madison, WI, USA). Cells were then infected with lentivirus to achieve appropriate knockdown without significant viral toxicity. Following selection, cell proliferation was assayed by CellTiter-Glo at 10 days. Independent experiments were repeated a minimum of three times in triplicates.

### Small molecule validation experiments

On day 0, cells were plated at densities optimized such that they would reach confluency after 5 days. On day 1, the specified compound was then added to each well at specified concentrations. KPT-330, LEE011 and palbociclib were obtained from Selleck Chemicals (Houston, TX, USA) and diluted in DMSO. Independent experiments were repeated a minimum of three times in triplicates.

### Cell cycle analysis

Cells were grown to subconfluency. Cells were trypsinized, fixed in 1% paraformaldehyde and then resuspended in 70% ethanol. Cells were treated with propidium iodide/RNAse (BD Biosciences, Franklin Lakes, NJ, USA) and flow cytometry performed on a BD LSR Fortessa where 10,000 events were assessed. These were then analyzed for cell cycle distribution by the ModFit LT (Verity Software House, Topsham, ME, USA).

### Immunoblots

After indicated treatments, cell lysates were harvested using RIPA buffer (Cell Signaling Technologies, Danvers, MA, USA) with protease inhibitors (cOmplete, Roche) and phosphatase inhibitors (PhosSTOP, Roche). Nuclear and cytoplasmic fractions were extracted using NE-PER (ThermoFisher Scientific). Antibodies used were as follows: CRM1 (XPO1; sc-5595; 1:5,000), CDK4 (sc-601; 1:2,000), cyclin D1 (sc-718; 1:500), beta-actin (sc-47778; 1:1,000), p53 (DO-1; sc-126; 1:500) from Santa Cruz Biotechnology and p21 (2947; 1:2,000), phospho-Rb S807/S811 (9308; 1:500), Rb (4H1; 9309P; 1:2,000), cleaved caspase-3 (9664; 1:1,000) from Cell Signaling Technologies. Uncropped scans are found in Supplementary Figs 7 and 8.

### *In vivo* tumour injections

This research project has been reviewed by the Dana-Farber Cancer Institute's Animal Care and Use Committee and approved under protocol 04-101, in compliance with the Animal Welfare Act and the Office of Laboratory Welfare (OLAW) of the National Institutes of Health (NIH). Overall, 5 × 10^6^ cells of CLF-PED-015-T in 100 μl of a 50% PBS/50% Matrigel (BD Biosciences) mixture were injected into flanks either unilaterally or bilaterally in Taconic NCr-Nude (CrTac:NCr-*Foxn1*^*nu*^) female mice at 6–8 weeks of age. Mice were dosed as followed for the *in vivo* study: palbociclib, 150 mg kg^−1^ daily by mouth; KPT-330, 15 mg kg^−1^ Monday/Wednesday/Friday by mouth; doxorubicin, 5 mg kg^−1^ weekly intravenously; palbociclib, 100 mg kg^−1^ daily and KPT-330, 15 mg kg^−1^ Monday/Wednesday/Friday. Dosing was held if mice lost weight>15% of day 1 weight. For BE(2)C, 1 × 10^6^ cells of a 50% PBS/50% Matrigel (BD Biosciences) mixture was used. For TC-32, 5 × 10^6^ cells of a 30% PBS/70% Matrigel (BD Biosciences) mixture was used. Mice were excluded if death occurred within the first 14 days of treatment. No randomization occurred. No blinding occurred.

### Micro-dosing device

Dosing was performed as previously described^21^. Compounds were formulated at 50% by weight in a matrix of PEG 1450 which is equivalent to ∼1 μg of compound per reservoir.

### Data availability

Sequencing data reported in this paper (whole-exome sequencing and RNA-sequencing) has been deposited in the database of Genotypes and Phenotypes (dbGaP) under study accession (phs001121.v1.p1). Noted plasmids in the text are available through Addgene or the Genomics Perturbations Platform at the Broad Institute of Harvard and MIT. CLF-PED-015-T cell line is available through the Cancer Cell Line Factory at the Broad Institute of Harvard and MIT. All primary data are available from the authors.

## Additional information

**How to cite this article**: Hong, A.L. *et al*. Integrated genetic and pharmacologic interrogation of rare cancers. *Nat. Commun.* 7:11987 doi: 10.1038/ncomms11987 (2016).

## Supplementary Material

Supplementary Information Supplementary Figures 1-8 and Supplementary Tables 1-13
