# Supplementary material for: Integrated genetic and pharmacologic interrogation of rare cancers
Source: Nat Commun. 2016 Jun 22;7:11987. doi: 10.1038/ncomms11987 (PMC4917959; doi:10.1038/ncomms11987)
Supplement: Supplementary Information — Supplementary Figures 1-8 and Supplementary Tables 1-13 [file ncomms11987-s1.pdf]

Supplementary Figure 1

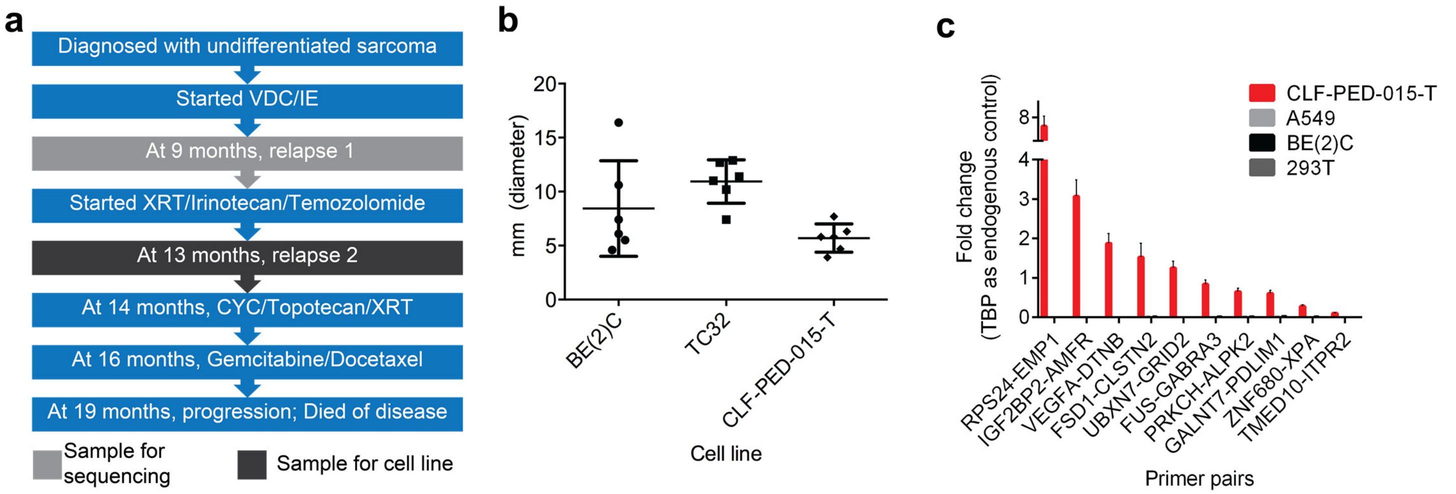

Supplementary Figure 1: Derivation and characterization of CLF-PED-015-T cell line. (a) Schematic of patient's clinical course. Sample for the patient's sequencing was obtained at the first relapse. The ell line was derived following the patient's second relapse. (b) Subcutaneous tumor size (by diameter) of CLF-PED-015-T in comparison to BE(2)C and TC32 (3 mice and 2 tumor sites per cell line) at 16 days. (c) Fold changes ($2^{-\Delta\Delta Ct}$) of novel fusions in comparison to a control gene, *TBP*. Three biological replicates were performed in triplicates. Error bars represent mean ±SD.

Supplementary Figure 2

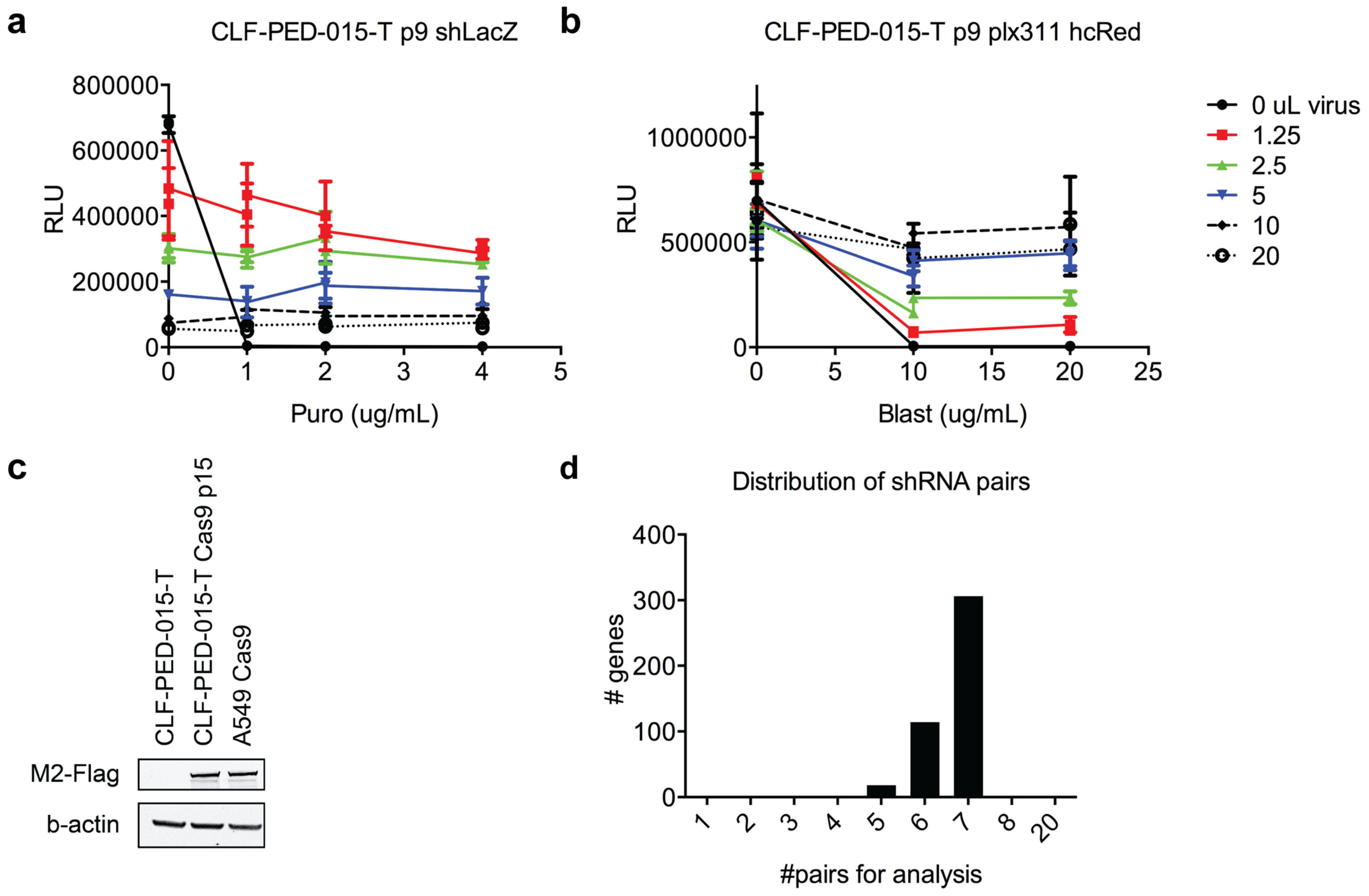

Supplementary Figure 2: Confirmation of lentiviral delivery into CLF-PED-015-T. (a-b) Determination of the multiplicity of infection. Titration of CLF-PED-015-T with lentiviral vectors containing a control shRNA targeting LacZ and the HcRed open reading frame (ORF). (c) Immunoblot detection of Cas9 expression with M2-Flag antibody. (d) Histogram of shRNA constructs used. Distribution of shRNA pairs used for analysis of the shRNA DCT v1.0 pool.

Supplementary Figure 3

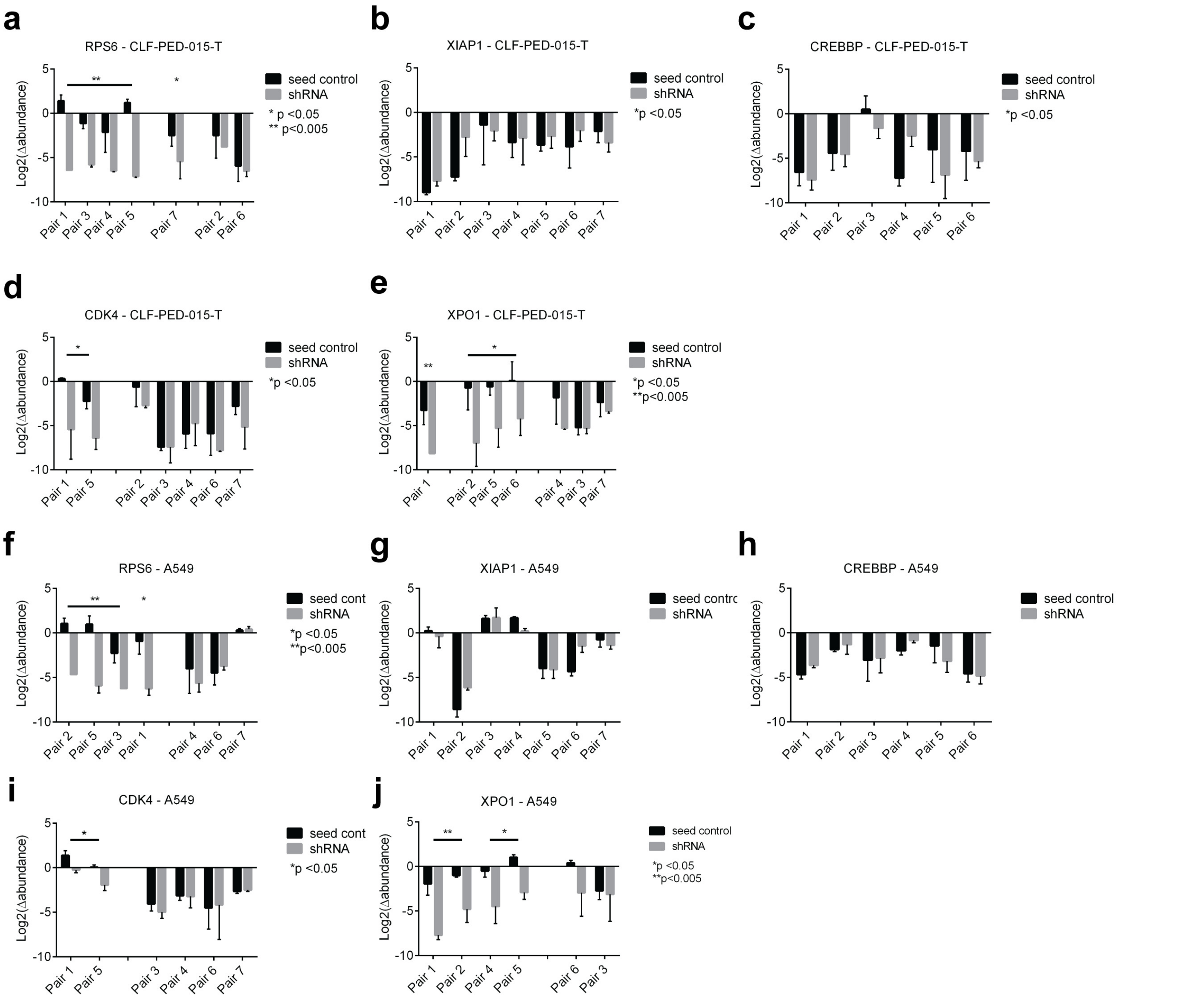

Supplementary Figure 3: Confirmation of shRNA on-target effects using seed controls. Analysis of shRNA DCT v1.0 pool with matched seed control based on log2 barcode reads. (a) Dependency on RPS6, a known essential gene in CLF-PED-015-T. (b-c) Seed effects prevent assessment of XIAP1 and CREBBP dependency in CLF-PED-015-T. (d-e) Dependency on XPO1 and CDK4 in CLF-PED-015-T. (f) Dependency on RPS6 in A549. (g-h) Seed effects prevent assessment of XIAP1 and CREBBP dependency in A549. (j) shRNA and seed control on CDK4 in A549. (j) Dependency on XPO1 in A549.

Supplementary Figure 4

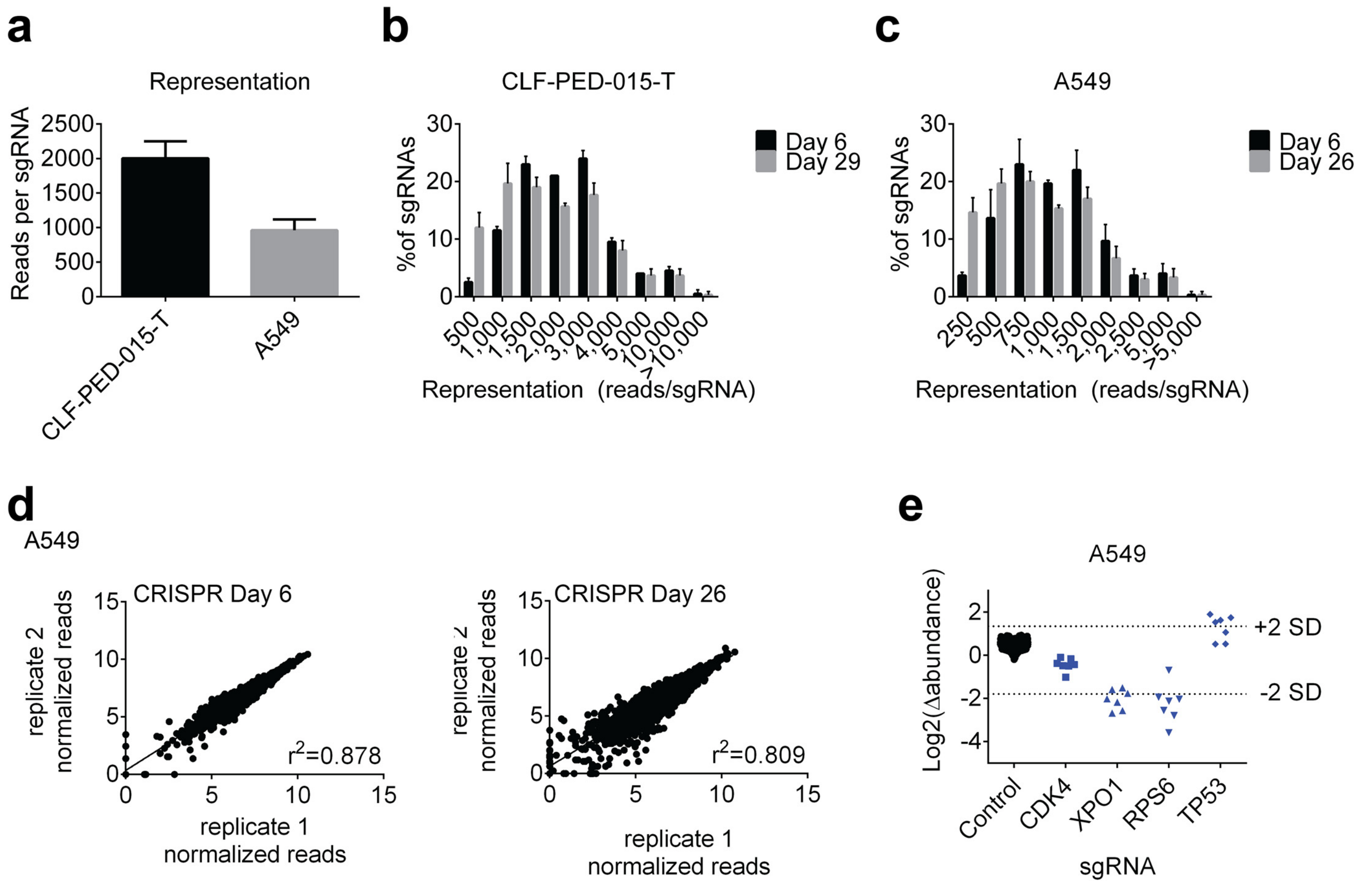

Supplementary Figure 4: CRISPR-Cas9 screening in CLF-PED-015-T feasible and comparable with A549. (a) Infection rate in CLF-PED-015-T based on reads per introduced sgRNA. (b-c) Representation of sgRNAs in CLF-PED-015-T at day 6 and at day 29 is comparable to A549. Error bars represent mean ±SD. (d) Correlation coefficients of replicates for CRISPR-Cas9 screens for A549 at days 6 and 26. (e) Consequences of deleting the indicated genes in A549.

Supplementary Figure 5

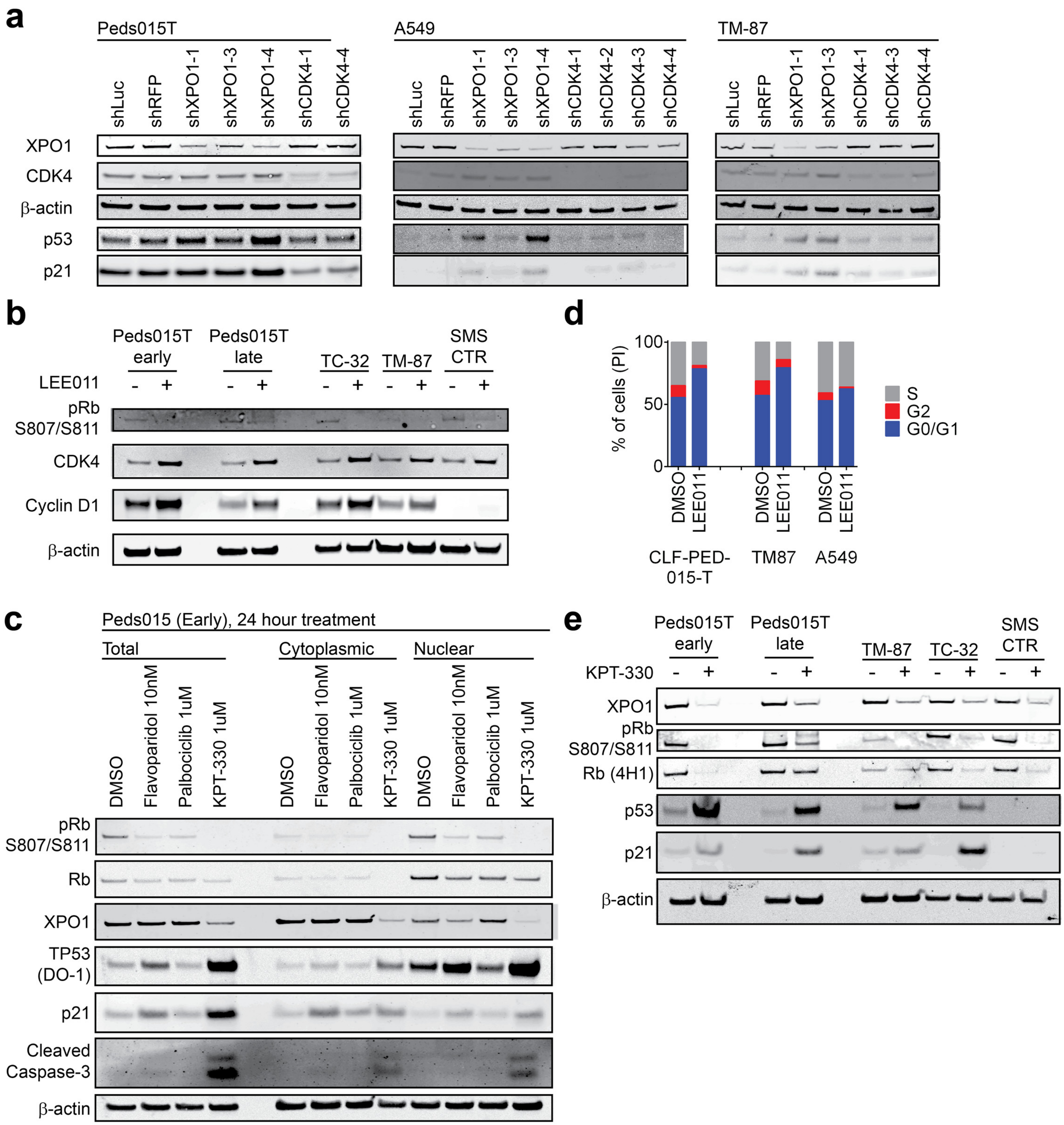

Supplementary Figure 5: Effects of CDK4 and XPO1 inhibitors. CDK4 inhibition leads to accumulation of cells in G0/1 and XPO1 inhibition leads to increased levels of p53 and cleaved caspase-3. a) Effects of XPO1-specific and CDK4-specific shRNAs. b) Phosphorylation of Rb (S807/S811) is inhibited and CDK4 is upregulated after treatment with the CDK4/6 inhibitor, LEE011. c) Effects of a pan-CDK inhibitor, flavopiridol, a CDK4/6 inhibitor, palbociclib and a XPO1 inhibitor, KPT-330, on Rb and p53 in nuclear and cytoplasmic fractions. KPT-330 led to increased TP53 and accumulation of cleaved caspase-3 in vitro. d) Treatment of CLF-PED-015-T with LEE011 results in a G0/G1 arrest. Data shown an average of two independent experiments. e) KPT-330 treatment results in an increase in total TP53 and inhibition of Rb.

Supplementary Figure 6

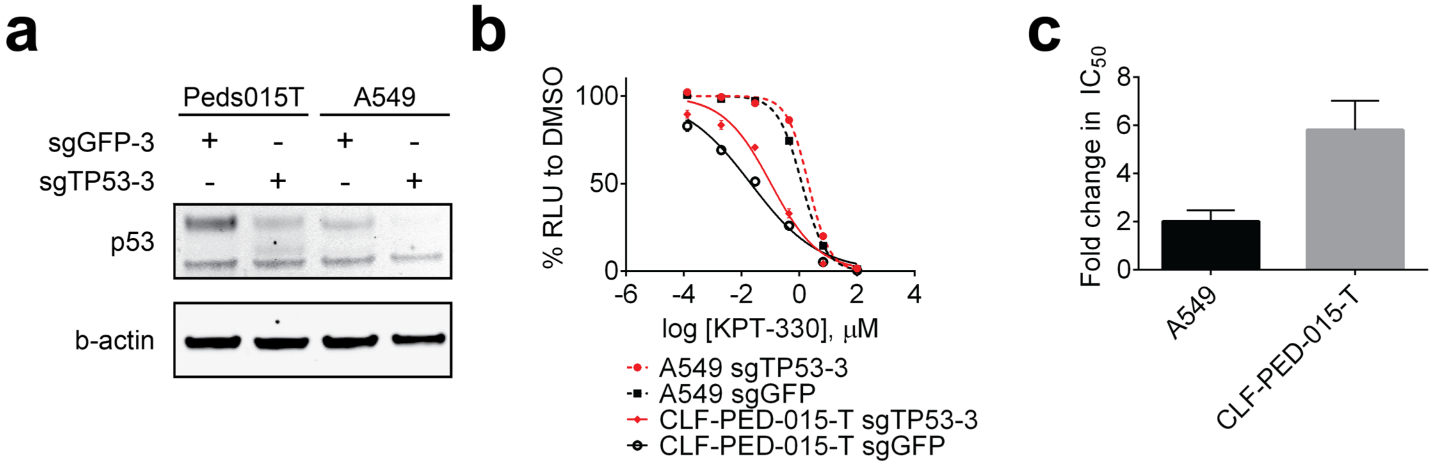

Supplementary Figure 6: Perturbation of TP53 causes a modest shift in the IC_{50}. We introduced sgRNAs targeting either GFP or TP53 in CLF-PED-015-T and A549 cells. (a) Decrease in p53 protein levels with sgRNAs compared to GFP controls. (b) Dose-response curves. Error bars represent mean ±SEM. (c) Data from (b) shown as a fold change compared to GFP controls. The fold change is based on biological duplicates with technical triplicates. Error bars represent mean ±SD.

Supplementary Figure 7 – uncropped blots

Supplementary Figure 2c:

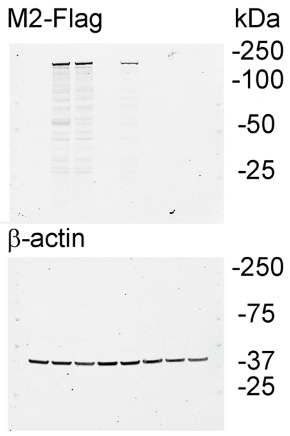

Supplementary Figure 5a:

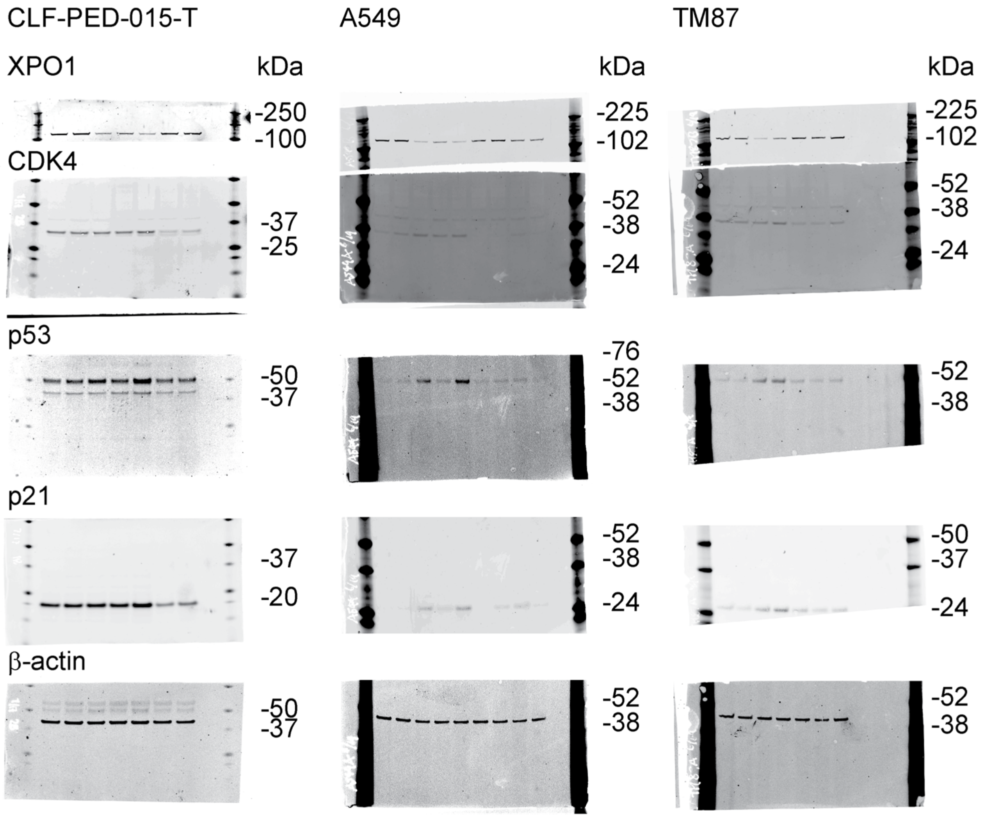

Supplementary Figure 5b:

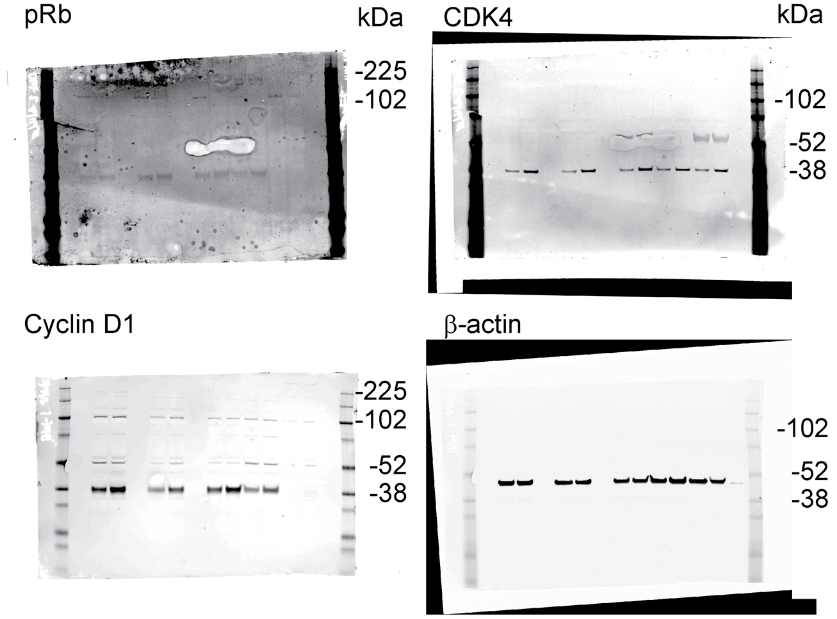

Supplementary Figure 5c:

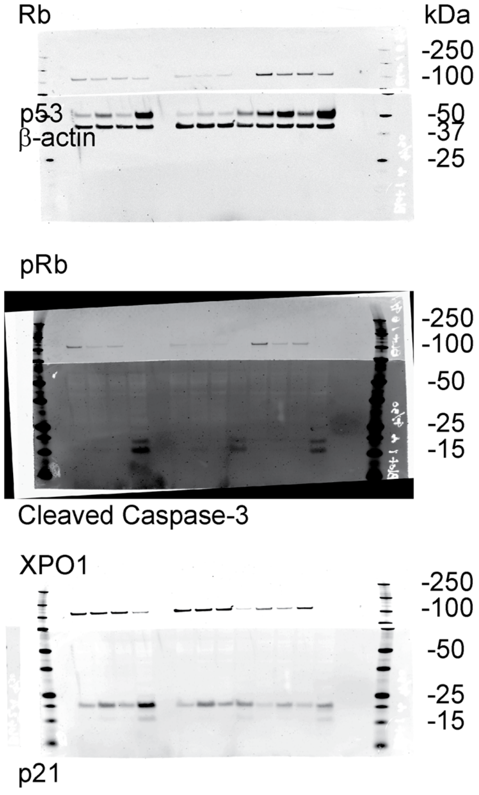

Supplementary Figure 8 – uncropped blots

Supplementary Figure 5e:

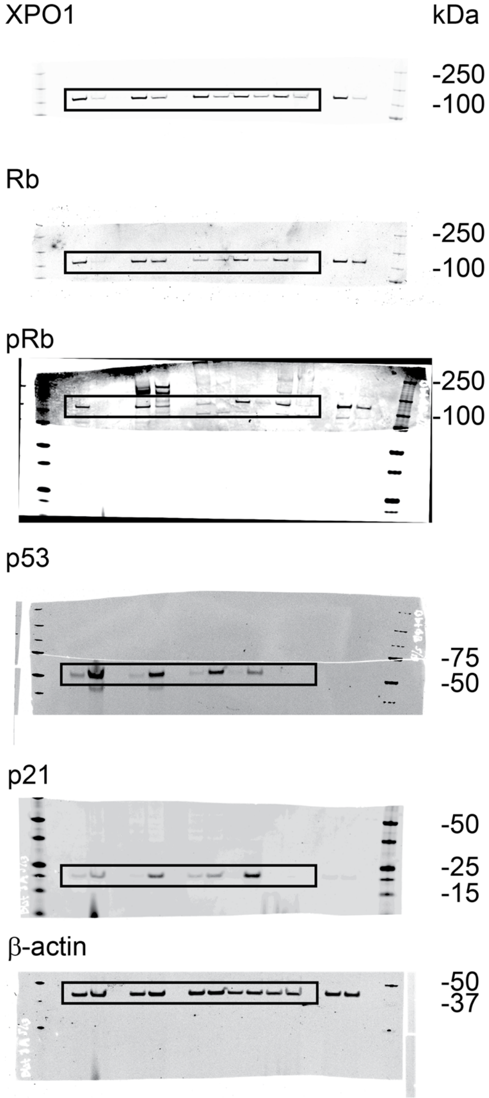

Supplementary Figure 6a:

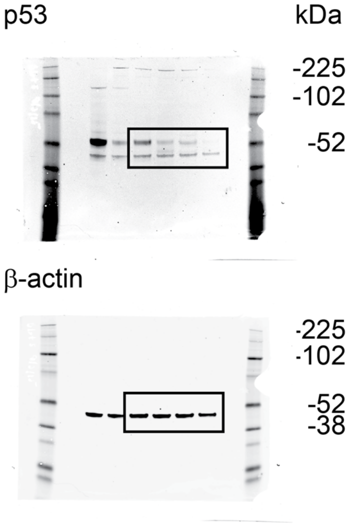

Supplementary Table 1: Listing of alterations seen between CLF-PED-015-T Tissue (at relapse 1) compared to cell line (at relapse 2)

Sample	Gene	Alteration	Variant_Classification	cDNA_Change	Allelic_fraction	Coverage
Metastatic Tissue	ASPA	p.D104G	Missense_Mutation	c.311A>G	24%	97
Metastatic Tissue	BAI3	p.C372R	Missense_Mutation	c.1114T>C	21%	104
Metastatic Tissue	C17orf97	p.DPEALKGFHPDPKALKGFHP318del	In_Frame_Del	c.952_1011delGACCCCGAGGCCCTCAAGGGCTTCCACCCCGACCCCAAGGCCCTCAAGGGCTTCCACCCC	29%	92
Metastatic Tissue	FAXDC2	p.Q61K	Missense_Mutation	c.181C>A	17%	42
Metastatic Tissue	GBF1	p.G814D	Missense_Mutation	c.2441G>A	20%	220
Metastatic Tissue	GGA2	p.C429F	Missense_Mutation	c.1286G>T	14%	94
Metastatic Tissue	GLTPD2	*Deleted	Copy Number			
Metastatic Tissue	INPP5K	*p.N128S	Missense_Mutation	c.383A>G	42%	50
Metastatic Tissue	ITGB2	*p.C462fs	Frame_Shift_Ins	c.1383_1384insC	20%	98
Metastatic Tissue	KIAA0226	*p.P696S	Missense_Mutation	c.2086C>T	27%	44
Metastatic Tissue	NIPSNAP3B	p.A222A	Splice_Site	c.666T>G	11%	38
Metastatic Tissue	PSMB6	*Deleted	Copy Number			
Metastatic Tissue	SIRPB2	*p.E45G	Missense_Mutation	c.134A>G	41%	64
Metastatic Tissue	SOBP	p.D58N	Missense_Mutation	c.172G>A	26%	114
Metastatic Tissue	SPRR3	p.EPGCTKVP95del	In_Frame_Del	c.163_186delGAGCCAGGCTGTACCAAGGTCCCT	15%	71
Metastatic Tissue	TBP	p.64_64Q>QQ	In_Frame_Ins	c.189_190insCAG	32%	96
Metastatic Tissue	THOC6	p.D154G	Missense_Mutation	c.461A>G	13%	40
Metastatic Tissue	TM4SF5	*Deleted	Copy Number			
Metastatic Tissue	VMO1	*Deleted	Copy Number			
Metastatic Tissue	XIRP1	p.L175V	Missense_Mutation	c.523C>G	12%	65
CLF-PED-015-T Cell Line	AFP	p.T16A	Missense_Mutation	c.46A>G	55%	51
CLF-PED-015-T Cell Line	ARID3B	p.R379fs	Frame_Shift_Ins	c.1133_1134insG	54%	70
CLF-PED-015-T Cell Line	ATM	p.QRH446fs	Frame_Shift_Del	c.1337_1343delAGCGACA	20%	95

CLF-PED-015-T Cell Line	BEGAIN	p.D33Y	Missense_Mutation	c.97G>T	15%	52
CLF-PED-015-T Cell Line	BLID	Amplified	Copy Number			
CLF-PED-015-T Cell Line	C15orf26	p.A196S	Missense_Mutation	c.586G>T	13%	68
CLF-PED-015-T Cell Line	C7	p.C782W	Missense_Mutation	c.2346T>G	15%	66
CLF-PED-015-T Cell Line	CNTNAP3	p.G331A	Missense_Mutation	c.992G>C	55%	67
CLF-PED-015-T Cell Line	COL12A1	p.I418K	Missense_Mutation	c.1253T>A	51%	119
CLF-PED-015-T Cell Line	DNAH17	p.E773K	Missense_Mutation	c.2317G>A	38%	66
CLF-PED-015-T Cell Line	FAM47C	p.G398V	Missense_Mutation	c.1193G>T	12%	52
CLF-PED-015-T Cell Line	GLTPD2	*Deleted	Copy Number			
CLF-PED-015-T Cell Line	GPR110	p.G887G	Splice_Site	c.2661C>A	10%	40
CLF-PED-015-T Cell Line	GSR	p.SS514del	In_Frame_Del	c.1539_1544delCTCTTC	44%	41
CLF-PED-015-T Cell Line	HGD	p.EE194fs	Frame_Shift_Del	c.582_585delGGAG	38%	86
CLF-PED-015-T Cell Line	IFRD1	p.K262R	Missense_Mutation	c.785A>G	45%	51
CLF-PED-015-T Cell Line	INPP5K	*p.N128S	Missense_Mutation	c.383A>G	43%	28
CLF-PED-015-T Cell Line	ITGB2	*p.C462fs	Frame_Shift_Ins	c.1383_1384insC	19%	48
CLF-PED-015-T Cell Line	KIAA0226	*p.P696S	Missense_Mutation	c.2086C>T	41%	22
CLF-PED-015-T Cell Line	LINGO2	p.D506H	Missense_Mutation	c.1516G>C	15%	68
CLF-PED-015-T Cell Line	LTF	p.L658R	Missense_Mutation	c.1973T>G	44%	186
CLF-PED-015-T Cell Line	MIR100	Amplified	Copy Number			

CLF-PED-015-T Cell Line	MIRLET7A2	Amplified	Copy Number			
CLF-PED-015-T Cell Line	MLLT6	p.D244Y	Missense_Mutation	c.730G>T	12%	50
CLF-PED-015-T Cell Line	MYT1L	p.P505L	Missense_Mutation	c.1514C>T	53%	78
CLF-PED-015-T Cell Line	N4BP1	p.V507G	Missense_Mutation	c.1520T>G	44%	102
CLF-PED-015-T Cell Line	PCYOX1L	p.F437S	Missense_Mutation	c.1310T>C	50%	42
CLF-PED-015-T Cell Line	PDCD10	p.L160V	Missense_Mutation	c.478C>G	29%	35
CLF-PED-015-T Cell Line	PHC2	p.N743S	Missense_Mutation	c.2228A>G	39%	38
CLF-PED-015-T Cell Line	PKP2	p.Q616E	Missense_Mutation	c.1846C>G	61%	36
CLF-PED-015-T Cell Line	PNPLA4	p.A103fs	Frame_Shift_Del	c.308delC	63%	35
CLF-PED-015-T Cell Line	POTEC	p.T93M	Missense_Mutation	c.278C>T	14%	108
CLF-PED-015-T Cell Line	PRB3	p.R242H	Missense_Mutation	c.725G>A	14%	22
CLF-PED-015-T Cell Line	PSMB6	*Deleted	Copy Number			
CLF-PED-015-T Cell Line	RIPK4	p.W579C	Missense_Mutation	c.1737G>T	10%	39
CLF-PED-015-T Cell Line	RNF17	p.P1298T	Missense_Mutation	c.3892C>A	44%	147
CLF-PED-015-T Cell Line	SCN7A	p.F1476fs	Frame_Shift_Del	c.4428delT	40%	35
CLF-PED-015-T Cell Line	SIK1	p.Q271H	Missense_Mutation	c.813G>T	24%	25
CLF-PED-015-T Cell Line	SIRPB2	*p.E45G	Missense_Mutation	c.134A>G	52%	23
CLF-PED-015-T Cell Line	SLC12A1	p.D758G	Missense_Mutation	c.2273A>G	35%	72
CLF-PED-015-T Cell Line	SNRNP35	p.E178G	Missense_Mutation	c.533A>G	36%	39

CLF-PED-015-T Cell Line	TM4SF5	*Deleted	Copy Number			
CLF-PED-015-T Cell Line	TTN	p.V16346M	Missense_Mutation	c.49036G>A	14%	22
CLF-PED-015-T Cell Line	VMO1	*Deleted	Copy Number			
CLF-PED-015-T Cell Line	ZNF235	p.H453Q	Missense_Mutation	c.1359C>A	55%	62

* Overlaps

Supplementary Table 2: Fusions common across Chimerascan, STAR and PRADA

**chrom5p**	**chrom3p**	**type**	**fusion_name**	**start5p.TP1**	**end5p.TP1**	**start3p.TP1**	**end3p.TP1**	**sample.TP1**
chr10	chr12	Interchromosomal	RPS24--EMP1	79793517	79795477	13366412	13369706	Metastatic specimen
chr14	chr12	Interchromosomal	TMED10--ITPR2	75643057	75643348	26488286	26564344	Metastatic specimen
chr14	chr18	Interchromosomal	PRKCH--ALPK2	61788514	61920070	56148483	56184413	Metastatic specimen
chr16	chrX	Interchromosomal	FUS--GABRA3	31191452	31196499	151335633	151514173	Metastatic specimen
chr3	chr16	Interchromosomal	IGF2BP2--AMFR	185542570	185542826	56395364	56448255	Metastatic specimen
chr3	chr4	Interchromosomal	UBXN7--GRID2	196089164	196159344	94006145	94138416	Metastatic specimen
chr4	chr10	Interchromosomal	GALNT7--PDLIM1	174089903	174090111	96997331	97007122	Metastatic specimen
chr6	chr2	Interchromosomal	VEGFA--DTNB	43737952	43746654	25606205	25611229	Metastatic specimen
chr7	chr9	Interchromosomal	ZNF680--XPA	64023302	64023504	100437191	100437868	Metastatic specimen
chrom5p	chrom3p	type	fusion_name	start5p	end5p	start3p	end3p	sample
chr10	chr12	Interchromosomal	RPS24--EMP1	79793517	79795477	13366412	13369706	CLF-PED-015-T
chr16	chrX	Interchromosomal	FUS--GABRA3	31191452	31196499	151335633	151514173	CLF-PED-015-T
chr3	chr4	Interchromosomal	UBXN7--GRID2	196088714	196159344	94006145	94138416	CLF-PED-015-T
chr4	chr10	Interchromosomal	GALNT7--PDLIM1	174089903	174090111	96997331	97007122	CLF-PED-015-T
chr6	chr2	Interchromosomal	VEGFA--DTNB	43737952	43749823	25606205	25611229	CLF-PED-015-T
chr19	chr3	Interchromosomal	FSD1--CLSTN2	4304590	4312047	140122470	140275896	CLF-PED-015-T
chr7	chr9	Interchromosomal	ZNF680--XPA	64004084	64023504	100437191	100437868	CLF-PED-015-T
chr3	chr16	Interchromosomal	IGF2BP2--AMFR	185540940	185542826	56395364	56443499	CLF-PED-015-T

Supplementary Table 3: Druggable targets (both shRNA and CRISPR)

Symbol	HGNC ID	Location
ABCG2	HGNC:74	4q22.1
ABL1	HGNC:76	9q34.1
ABL2	HGNC:77	1q25.2
ACAT1	HGNC:93	11q22.3
ACER1	HGNC:18356	19p13.3
ACER2	HGNC:23675	9p21.3
ACER3	HGNC:16066	11q13.5
ACTB	HGNC:132	7p22
ADORA2A	HGNC:263	22q11.23
AGXT	HGNC:341	2q37.3
AHR	HGNC:348	7p15
AKT1	HGNC:391	14q32.33
AKT2	HGNC:392	19q13.1-q13.2
AKT3	HGNC:393	1q44
ALK	HGNC:427	2p23
ANPEP	HGNC:500	15q25-q26
APC	HGNC:583	5q21-q22
APH1A	HGNC:29509	1q21.2
AR	HGNC:644	Xq12
ARAF	HGNC:646	Xp11.3-p11.23
ARF1	HGNC:652	1q42.13
ARFGAP1	HGNC:15852	20q13.33
ARFGAP3	HGNC:661	22q13.2
ARG1	HGNC:663	6q23
ARG2	HGNC:664	14q24.1
ASAH1	HGNC:735	8p22
ASAH2	HGNC:18860	10q11.23
ATM	HGNC:795	11q22-q23
ATP1A1	HGNC:799	1p13
ATP1A2	HGNC:800	1q23.2
ATP1A3	HGNC:801	19q13.2
ATP1A4	HGNC:14073	1q23.2
ATP1B1	HGNC:804	1q24.2
ATP1B3	HGNC:806	3q23
ATP1B4	HGNC:808	Xq24
ATP5L2	HGNC:13213	22q13.2
ATP6V0A1	HGNC:865	17q21
ATR	HGNC:882	3q23
AURKA	HGNC:11393	20q13
AURKB	HGNC:11390	17p13.1
AURKC	HGNC:11391	19q13.43
AXIN1	HGNC:903	16p13.3
AXL	HGNC:905	19q13.1
BAX	HGNC:959	19q13.3-q13.4
BCL2	HGNC:990	18q21.3
BCL2L1	HGNC:992	20q11.21
BCL2L10	HGNC:993	15q21
BCL2L2	HGNC:995	14q11.2-q12

BCR	HGNC:1014	22q11
BIRC2	HGNC:590	11q22
BIRC3	HGNC:591	11q22
BIRC5	HGNC:593	17q25.3
BRAF	HGNC:1097	7q34
BRD2	HGNC:1103	6p21.3
BRD3	HGNC:1104	9q34
BRD4	HGNC:13575	19p13.12
BRDT	HGNC:1105	1p22.1
BTK	HGNC:1133	Xq21.33-q22
CA1	HGNC:1368	8q21.2
CA12	HGNC:1371	15q22
CA13	HGNC:14914	8q21.2
CA14	HGNC:1372	1q21
CA2	HGNC:1373	8q21.2
CA3	HGNC:1374	8q21.2
CA4	HGNC:1375	17q23.1
CA6	HGNC:1380	1p36.2
CA7	HGNC:1381	16q22.1
CA9	HGNC:1383	9p13.3
CASP3	HGNC:1504	4q34
CASP6	HGNC:1507	4q25
CASP7	HGNC:1508	10q25
CCR4	HGNC:1605	3p24-p21.3
CD19	HGNC:1633	16p11.2
CD22	HGNC:1643	19q13.1
CD248	HGNC:18219	11q13
CD33	HGNC:1659	19q13.3
CD37	HGNC:1666	19q13.33
CD38	HGNC:1667	4p15.32
CD52	HGNC:1804	1p36
CD79B	HGNC:1699	17q23
CDA	HGNC:1712	1p36.2-p35
CDC25A	HGNC:1725	3p21
CDC25B	HGNC:1726	20p13
CDC25C	HGNC:1727	5q31
CDK1	HGNC:1722	10q21.2
CDK2	HGNC:1771	12q13
CDK4	HGNC:1773	12q13
CDK5	HGNC:1774	7q36
CDK6	HGNC:1777	7q21-q22
CDK7	HGNC:1778	5q12.1
CDK9	HGNC:1780	9q34.1
CEACAM5	HGNC:1817	19q13.1-q13.2
CERK	HGNC:19256	22q13.31
CERS1	HGNC:14253	19p12
CERS2	HGNC:14076	1q21.3
CERS3	HGNC:23752	15q26.3
CERS4	HGNC:23747	19p13.2
CERS5	HGNC:23749	12q13.12

CERS6	HGNC:23826	2q31
CHEK1	HGNC:1925	11q24.2
CHEK2	HGNC:16627	22q12.1
CLK1	HGNC:2068	2q33
CLK4	HGNC:13659	5q35
CLTA	HGNC:2090	9p13.3
CLTB	HGNC:2091	5q35.2
CLTC	HGNC:2092	17q23.1
CLTCL1	HGNC:2093	22q11.2
CMPK1	HGNC:18170	1p34.1-p33
CPT1A	HGNC:2328	11q13.2
CRBN	HGNC:30185	3p26.3
CREB1	HGNC:2345	2q34
CREBBP	HGNC:2348	16p13.3
CSF1R	HGNC:2433	5q32
CSNK1A1	HGNC:2451	5q32
CSNK1D	HGNC:2452	17q25
CSNK1E	HGNC:2453	22q13.1
CSNK2A1	HGNC:2457	20p13
CSNK2A2	HGNC:2459	16q21
CTLA4	HGNC:2505	2q33
CTNNB1	HGNC:2514	3p21
CUL1	HGNC:2551	7q36.1
CUL4A	HGNC:2554	13q34
CUL4B	HGNC:2555	Xq23
CXCR2	HGNC:6027	2q35
CXCR4	HGNC:2561	2q21
CYP17A1	HGNC:2593	10q24.3
CYP19A1	HGNC:2594	15q21
DDB1	HGNC:2717	11q12.2
DHFR	HGNC:2861	5q14.1
DIABLO	HGNC:21528	12q24.31
DLL4	HGNC:2910	15q14
DNM1	HGNC:2972	9q34
DNMT1	HGNC:2976	19p13.2
DOT1L	HGNC:24948	19p13.3
DPP4	HGNC:3009	2q24.2
DRD2	HGNC:3023	11q23.2
EDNRB	HGNC:3180	13q22
EGFR	HGNC:3236	7p12
EHMT1	HGNC:24650	9q34.3
EHMT2	HGNC:14129	6p21.3
ENPP3	HGNC:3358	6q22
EP300	HGNC:3373	22q13.2
EPCAM	HGNC:11529	2p21
EPHA2	HGNC:3386	1p36
ERBB2	HGNC:3430	17q11.2-q12
ERBB3	HGNC:3431	12q13
ERBB4	HGNC:3432	2q33.3-q34
ERG	HGNC:3446	21q22.3

ESR1	HGNC:3467	6q24-q27
ESR2	HGNC:3468	14q21-q22
ETV1	HGNC:3490	7p22
EWSR1	HGNC:3508	22q12.2
EZH2	HGNC:3527	7q35-q36
F2R	HGNC:3537	5q13
FAAH	HGNC:3553	1p35-p34
FASN	HGNC:3594	17q25
FGFR1	HGNC:3688	8p11.23-p11.22
FGFR2	HGNC:3689	10q25.3-q26
FGFR3	HGNC:3690	4p16.3
FGFR4	HGNC:3691	5q35.2
FLI1	HGNC:3749	11q24.1-q24.3
FLT1	HGNC:3763	13q12
FLT3	HGNC:3765	13q12
FLT4	HGNC:3767	5q34-q35
FNTA	HGNC:3782	8p11.21
FNTB	HGNC:3785	14q23.3
FOLH1	HGNC:3788	11p11.2
FYN	HGNC:4037	6q21
FZD7	HGNC:4045	2q33
GLI1	HGNC:4317	12q13.3
GLS	HGNC:4331	2q32-q34
GM2A	HGNC:4367	5q33.1
GNRH1	HGNC:4419	8p21-p11.2
GSK3A	HGNC:4616	19q13
GSK3B	HGNC:4617	3q13.3
GUCY2C	HGNC:4688	12p12
HDAC1	HGNC:4852	1p34
HDAC10	HGNC:18128	22q13.31
HDAC11	HGNC:19086	3p25.1
HDAC2	HGNC:4853	6q21
HDAC3	HGNC:4854	5q31.1-q31.2
HDAC4	HGNC:14063	2q37.3
HDAC5	HGNC:14068	17q21
HDAC6	HGNC:14064	Xp11.23
HDAC7	HGNC:14067	12q13.1
HDAC8	HGNC:13315	Xq13
HDAC9	HGNC:14065	7p21.1
HGF	HGNC:4893	7q21.1
HMGCR	HGNC:5006	5q13.3-q14
HMGCS1	HGNC:5007	5p12
HNF4A	HGNC:5024	20q13.12
HRH2	HGNC:5183	5q35
HSP90AA1	HGNC:5253	14q32.33
HSP90AA2	HGNC:5256	11p14.1
HSP90AB1	HGNC:5258	6p12
HSP90B1	HGNC:12028	12q24.2-q24.3
HSPA1A	HGNC:5232	6p21.3
HSPA1B	HGNC:5233	6p21.3

HSPA1L	HGNC:5234	6p21.3
IDH1	HGNC:5382	2q34
IFNA2	HGNC:5423	9p22
IGF1	HGNC:5464	12q23.2
IGF1R	HGNC:5465	15q26.3
IGF2	HGNC:5466	11p15.5
IKBKB	HGNC:5960	8p11.2
IKBKE	HGNC:14552	1q32.1
IL12RB1	HGNC:5971	19p13.1
IL21	HGNC:6005	4q26-q27
IL6	HGNC:6018	7p15.3
INSR	HGNC:6091	19p13.3-p13.2
ITGA2	HGNC:6137	5q11.2
JAK1	HGNC:6190	1p32.3-p31.3
JAK2	HGNC:6192	9p24
JAK3	HGNC:6193	19p13-p12
JMJD6	HGNC:19355	17q25
KDM1A	HGNC:29079	1p36.12
KDM3A	HGNC:20815	2p11.2
KDM4A	HGNC:22978	1p34.1
KDM4B	HGNC:29136	19p13.3
KDM4C	HGNC:17071	9p24-p23
KDM4D	HGNC:25498	11q21
KDM6A	HGNC:12637	Xp11.2
KDM6B	HGNC:29012	17p13.1
KDR	HGNC:6307	4q11-q12
KIF11	HGNC:6388	10q24.1
KIT	HGNC:6342	4q12
KLF5	HGNC:6349	13q21.3
KPNB1	HGNC:6400	17q21.32
KRAS	HGNC:6407	12p12.1
LAP3	HGNC:18449	4p15.33
LCK	HGNC:6524	1p34.3
LRRK2	HGNC:18618	12q12
LYPLA1	HGNC:6737	8q11.23
MAGEA3	HGNC:6801	Xq28
MAP2K1	HGNC:6840	15q22.1-q22.33
MAP2K2	HGNC:6842	19p13.3
MAP3K8	HGNC:6860	10p11.2
MAPK10	HGNC:6872	4q22-q23
MAPK14	HGNC:6876	6p21.3-p21.2
MAPK3	HGNC:6877	16p11.2
MAPK8	HGNC:6881	10q11
MAPK9	HGNC:6886	5q35
MAPKAP1	HGNC:18752	9q34.11
MCL1	HGNC:6943	1q21
MDM2	HGNC:6973	12q13-q14
MEN1	HGNC:7010	11q13
MET	HGNC:7029	7q31
MGMT	HGNC:7059	10q26

MLST8	HGNC:24825	16p13.3
MMP1	HGNC:7155	11q22.2
MS4A1	HGNC:7315	11q12.2
MSLN	HGNC:7371	16p13.3
MTOR	HGNC:3942	1p36
MUC16	HGNC:15582	19p13.2
MYC	HGNC:7553	8q24
MYH1	HGNC:7567	17p13.1
MYH2	HGNC:7572	17p13.1
NAE1	HGNC:621	16q22
NAMPT	HGNC:30092	7q22.3
NCEH1	HGNC:29260	3q26.31
NCSTN	HGNC:17091	1q22-q23
NEDD8	HGNC:7732	14q11.2
NEUROD1	HGNC:7762	2q32
NEUROD2	HGNC:7763	17q12
NFKB1	HGNC:7794	4q24
NOTCH1	HGNC:7881	9q34.3
NOTCH2	HGNC:7882	1p13-p11
NOTCH3	HGNC:7883	19p13.12
NOTCH4	HGNC:7884	6p21.3
NPEPPS	HGNC:7900	17q12-q21
NR1D1	HGNC:7962	17q11.2
NR1H4	HGNC:7967	12q23.1
NR3C1	HGNC:7978	5q31-q32
P2RX7	HGNC:8537	12q24
PAK4	HGNC:16059	19q13.2
PARP1	HGNC:270	1q41-q42
PARP2	HGNC:272	14q11.2
PDE4A	HGNC:8780	19p13.2
PDE4B	HGNC:8781	1p31
PDE4D	HGNC:8783	5q12
PDE5A	HGNC:8784	4q27
PDGFRA	HGNC:8803	4q12
PDGFRB	HGNC:8804	5q33.1
PDK2	HGNC:8810	17q21.33
PI4KB	HGNC:8984	1q21
PICK1	HGNC:9394	22q13.1
PIK3CA	HGNC:8975	3q26.3
PIK3CB	HGNC:8976	3q22.3
PIK3CD	HGNC:8977	1p36.2
PIK3CG	HGNC:8978	7q22
PIM1	HGNC:8986	6p21
PLA2G4A	HGNC:9035	1q31.1
PLA2G4B	HGNC:9036	15q11.2-q21.3
PLA2G4C	HGNC:9037	19q13.3
PLA2G4D	HGNC:30038	15q14
PLAUR	HGNC:9053	19q13
PLD1	HGNC:9067	3q26
PLD2	HGNC:9068	17p13.3

PLK1	HGNC:9077	16p
PLK3	HGNC:2154	1p34.1
POLA1	HGNC:9173	Xp22.1-p21.3
POLA2	HGNC:30073	11q13
POLE	HGNC:9177	12q24.3
PORCN	HGNC:17652	Xp11.23
PPID	HGNC:9257	4q31.3
PPP2CA	HGNC:9299	5q31.1
PPP3CA	HGNC:9314	4q24
PPP3CB	HGNC:9315	10q22.2
PPP3CC	HGNC:9316	8p21.3
PPP3R1	HGNC:9317	2p14
PPP3R2	HGNC:9318	9q31
PRKCB	HGNC:9395	16p12
PRKCI	HGNC:9404	3q26.3
PRKCSH	HGNC:9411	19p13.2
PRKDC	HGNC:9413	8q11
PRLR	HGNC:9446	5p14-p13
PSEN1	HGNC:9508	14q24.3
PSENEN	HGNC:30100	19q13.12
PSMB1	HGNC:9537	6q27
PSMB2	HGNC:9539	1p34.2
PSMB5	HGNC:9542	14q11.2
PSMD1	HGNC:9554	2q37.1
PSMD2	HGNC:9559	3q27.3
PTGER3	HGNC:9595	1p31.2
PTGER4	HGNC:9596	5p13.1
PTGS2	HGNC:9605	1q25.2-q25.3
PTK2	HGNC:9611	8q24.3
PTP4A3	HGNC:9636	8q24.3
PVRL4	HGNC:19688	1q23.3
RAC1	HGNC:9801	7p22
RAD51	HGNC:9817	15q15.1
RAF1	HGNC:9829	3p25
RAN	HGNC:9846	12q24.33
RARA	HGNC:9864	17q21.1
RARB	HGNC:9865	3p24
RARG	HGNC:9866	12q13
RASA1	HGNC:9871	5q13
RET	HGNC:9967	10q11.2
RHOA	HGNC:667	3p21.3
RICTOR	HGNC:28611	5p13.1
RIPK1	HGNC:10019	6p25.2
RORA	HGNC:10258	15q21-q22
RORC	HGNC:10260	1q21
ROS1	HGNC:10261	6q21-q22
RPS6KB1	HGNC:10436	17q23.1
RPS6KB2	HGNC:10437	11q13.1
RPTOR	HGNC:30287	17q25.3
RRM1	HGNC:10451	11p15.5

RXRA	HGNC:10477	9q34
RXRB	HGNC:10478	6p21.3
RXRG	HGNC:10479	1q22-q23
S1PR1	HGNC:3165	1p21
S1PR3	HGNC:3167	9q22.1-q22.2
SCARB1	HGNC:1664	12q24.32
SHH	HGNC:10848	7q36
SIRT1	HGNC:14929	10q21
SIRT2	HGNC:10886	19q13
SIRT3	HGNC:14931	11p15.5
SIRT4	HGNC:14932	12q24.31
SIRT5	HGNC:14933	6p23
SIRT6	HGNC:14934	19p13.3
SIRT7	HGNC:14935	17q25.3
SKP1	HGNC:10899	5q31
SLC2A1	HGNC:11005	1p34.2
SLC34A2	HGNC:11020	4p15.2
SLITRK6	HGNC:23503	13q31.1
SMO	HGNC:11119	7q32.1
SNAP25	HGNC:11132	20p12-p11.2
SP1	HGNC:11205	12q13.1
SPHK1	HGNC:11240	17q25.2
SPTLC1	HGNC:11277	9q22.31
SPTLC2	HGNC:11278	14q24.3
SPTLC3	HGNC:16253	20p12.1
SRC	HGNC:11283	20q12-q13
SSTR1	HGNC:11330	14q13
SSTR3	HGNC:11332	22q13.1
SSTR5	HGNC:11334	16p13.3
STAT3	HGNC:11364	17q21
STAT5B	HGNC:11367	17q11.2
STEAP1	HGNC:11378	7q21
STK11	HGNC:11389	19p13.3
STX1A	HGNC:11433	7q11.2
SYK	HGNC:11491	9q22
TACC3	HGNC:11524	4p16.3
TASP1	HGNC:15859	20p12
TEK	HGNC:11724	9p21
TERT	HGNC:11730	5p15.33
TGFBR1	HGNC:11772	9q22
TGFBR2	HGNC:11773	3p22
TIAM1	HGNC:11805	21q22.1
TNF	HGNC:11892	6p21.3
TNFRSF4	HGNC:11918	1p36
TNFRSF8	HGNC:11923	1p36
TNFRSF9	HGNC:11924	1p36
TNFSF13B	HGNC:11929	13q32-q34
TNKS	HGNC:11941	8p23.1
TOP1	HGNC:11986	20q12-q13.1
TOP2A	HGNC:11989	17q21.2

TOP2B	HGNC:11990	3p24.2
TP53	HGNC:11998	17p13.1
TRIO	HGNC:12303	5p14-p15.1
TRPC6	HGNC:12338	11q22.1
TRPV4	HGNC:18083	12q24.11
TSPO	HGNC:1158	22q13.3
TTK	HGNC:12401	6q14.1
TUBB1	HGNC:16257	20q13.32
TXN	HGNC:12435	9q31
TYMS	HGNC:12441	18p11.31-p11.21
UBA1	HGNC:12469	Xp11.23
UCHL5	HGNC:19678	1q32
UGCG	HGNC:12524	9q31
USP10	HGNC:12608	16q24.1
USP13	HGNC:12611	3q26.2-q26.3
USP14	HGNC:12612	18p11.32
USP5	HGNC:12628	12p13
USP7	HGNC:12630	16p13.3
USP9X	HGNC:12632	Xp11.4
VAMP1	HGNC:12642	12p
VCP	HGNC:12666	9p13.3
VDAC1	HGNC:12669	5q31
VDAC2	HGNC:12672	10q22
VDR	HGNC:12679	12q13.11
VEGFA	HGNC:12680	6p12
WEE1	HGNC:12761	11p15.4
WRN	HGNC:12791	8p12
WT1	HGNC:12796	11p13
XIAP	HGNC:592	Xq25
XPO1	HGNC:12825	2p15
YES1	HGNC:12841	18p11.31-p11.21

Essential Genes

**Gene**	**ID**	**Location**
RPL10	HGNC:10298	Xq28
RPL7	HGNC:10363	8q13.3
RPS6	HGNC:10429	9p21
SFPQ	HGNC:10774	1p34.3
EIF5B	HGNC:30793	2q11.2
EIF3G	HGNC:3274	19p13.2

Supplementary Table 4: CLF-PED-015-T shRNA with seed control - hits as identified by RIGER

Gene	NES	p-value	# Hairpins 500	shRNA Gene Rank
RPS6	0.004545	0.0002	5	Essential gene
RPL7	0.01212	0.0006	5	Essential gene
RAN	0.01818	0.0011	3	1
UBA1	0.02045	0.0018	4	2
PSMD2	0.02197	0.0021	3	3
POLA1	0.0303	0.0036	4	4
EIF3G	0.03258	0.0044	2	Essential gene
CDK1	0.03485	0.0046	3	5
KIF11	0.0447	0.0079	6	6
XPO1	0.04545	0.0082	5	7
EIF5B	0.0553	0.0127	2	Essential gene
NR1D1	0.06386	0.0133	2	8
HDAC2	0.05606	0.0134	3	9
SFPQ	0.06364	0.0166	5	Essential gene
USP5	0.07164	0.017	2	10
PI4KB	0.07424	0.021	2	11
CSNK1A1	0.07576	0.0214	2	12
KLF5	0.0856	0.0225	3	13
PSMB5	0.08106	0.0251	3	14
RAD51	0.09103	0.026	3	15
RPL10	0.09511	0.0281	3	Essential gene
CDK4	0.09242	0.0318	3	16
WEE1	0.1012	0.0325	4	17
WRN	0.09394	0.0325	2	18
DHFR	0.09773	0.0352	2	19
PSMD1	0.1045	0.041	4	20
TXN	0.1068	0.0421	4	21

Supplementary Table 5: A549 shRNA with seed control - hits as identified by RIGER

Gene	NES	p-value	# Hairpins 500	shRNA Gene Rank
RPS6	0.01138	0.0009	5	Essential gene
VCP	0.01214	0.001	3	1
PSMB5	0.01551	0.001	2	2
SFPQ	0.01442	0.0013	5	Essential gene
EIF3G	0.02049	0.0023	3	Essential gene
MTOR	0.02807	0.0024	3	3
PSMD1	0.02656	0.0035	5	4
PSMD2	0.02731	0.0037	5	5
CDK1	0.02807	0.0041	4	6
AURKB	0.03304	0.0045	4	7
EWSR1	0.03978	0.0062	4	8
UBA1	0.04173	0.0066	4	9
HSP90B1	0.04249	0.0067	4	10
XPO1	0.04316	0.0069	5	11
KIF11	0.04932	0.009	3	12
BRD4	0.05311	0.0103	5	13
POLE	0.05387	0.011	2	14
NEDD8	0.05463	0.0113	2	15
CSNK1A1	0.06146	0.0141	4	16
CLTC	0.06677	0.0161	4	17
TACC3	0.07208	0.0183	2	18
KPNB1	0.0736	0.0189	4	19
CA6	0.07436	0.0197	2	20
RPTOR	0.07511	0.0202	2	21
RPL7	0.07891	0.0223	7	Essential gene
DDB1	0.08118	0.0243	4	22
PSMB1	0.08801	0.0279	5	23
POLA1	0.08953	0.0288	5	24
SPTLC2	0.09105	0.0301	2	25
USP7	0.0971	0.0304	4	26
ERBB2	0.09181	0.0306	5	27
RRM1	0.09408	0.032	3	28
RAD51	0.1005	0.0321	3	29
RAN	0.09788	0.0338	4	30
PTK2	0.09939	0.0344	4	31
TTK	0.1032	0.0349	3	32
PI4KB	0.1024	0.036	3	33
VDR	0.1039	0.0366	3	34
BCL2L1	0.1062	0.0382	4	35
CUL1	0.1092	0.0397	3	36

Supplementary Table 6: CLF-PED-015-T CRISPR hits as identified by RIGER

Gene	NES	p-value	# Hairpins 500	CRISPR Gene Rank
SP1	0.004428	0.0002	4	1
RAN	0.008856	0.0003	7	2
RPS6	0.009594	0.0003	7	Essential gene
USP5	0.01402	0.001	6	3
TOP2A	0.0155	0.0013	6	4
RPL7	0.01771	0.0019	5	Essential gene
CSNK1D	0.02583	0.0041	4	5
UBA1	0.02804	0.0047	5	6
POLE	0.02952	0.0048	6	7
WEE1	0.03026	0.0049	4	8
CDK4	0.03173	0.0053	5	9
MYC	0.03838	0.0067	6	10
KPNB1	0.04354	0.0087	5	11
KIF11	0.04428	0.0091	5	12
USP7	0.04723	0.0104	6	13
STK11	0.05314	0.0131	7	14
USP9X	0.05387	0.0132	6	15
RPTOR	0.05535	0.0139	5	16
HMGCS1	0.06421	0.0192	6	17
RRM1	0.06716	0.0197	6	18
ACTB	0.06863	0.0206	5	19
DHFR	0.07085	0.0213	4	20
CSNK1A1	0.07454	0.023	4	21
FNTA	0.07675	0.024	4	22
EIF3G	0.07823	0.0247	6	Essential gene
PSMB5	0.08339	0.0281	4	23
EWSR1	0.08487	0.029	4	24
PLK1	0.09004	0.0319	5	25
XPO1	0.09151	0.0324	6	26
BRD4	0.09299	0.0331	6	27
DDB1	0.09446	0.0342	4	28
CMPK1	0.0952	0.0348	4	29
SFPQ	0.1033	0.0405	6	Essential gene
TACC3	0.1085	0.0435	4	30
MDM2	0.1107	0.0448	4	31
VCP	0.1137	0.0472	4	32
AR	1.91	1	0	
TP53	2.51	1	0	
GNRH1	2.02	1	0	
CA13	2.03	1	0	

Supplementary Table 7: Comparison of shRNA and CRISPR hits for CLF-PED-015-T

Gene	shRNA Gene Rank	CRISPR Gene Rank
RPS6	Essential gene	Essential gene
RPL7	Essential gene	Essential gene
RAN	1	2
UBA1	2	6
PSMD2	3	
POLA1	4	
EIF3G	Essential gene	Essential gene
CDK1	5	
KIF11	6	12
XPO1	7	26
EIF5B	Essential gene	
NR1D1	8	
HDAC2	9	
SFPQ	Essential gene	Essential gene
USP5	10	3
PI4KB	11	
CSNK1A1	12	21
KLF5	13	
PSMB5	14	23
RAD51	15	
RPL10	Essential gene	
CDK4	16	9
WEE1	17	8
WRN	18	
DHFR	19	20
PSMD1	20	
TXN	21	

Supplementary Table 8: A549 CRISPR hits as identified by RIGER

Gene	NES	p-value	# Hairpins 500	CRISPR Gene Rank
UBA1	0.00369	0.0002	7	1
CDK7	0.005166	0.0002	6	2
USP5	0.009594	0.0003	6	3
RAN	0.01624	0.0014	6	4
RPL7	0.02214	0.0031	7	Essential gene
MYC	0.02288	0.0035	7	5
ATR	0.02362	0.0037	5	6
WEE1	0.02509	0.0039	4	7
DDB1	0.02804	0.0047	7	8
HMGCS1	0.03247	0.0053	6	9
USP7	0.03616	0.0059	6	10
VCP	0.0369	0.0062	7	11
RPS6	0.04133	0.0075	6	Essential gene
SFPQ	0.04354	0.0087	7	Essential gene
ACTB	0.04576	0.0097	7	12
CUL1	0.04871	0.0111	5	13
NEDD8	0.05092	0.0115	6	14
PSMB5	0.05166	0.0122	6	15
RPTOR	0.05535	0.0139	7	16
MTOR	0.05609	0.0141	7	17
TOP2A	0.05683	0.0144	6	18
FNTB	0.05904	0.0157	7	19
XPO1	0.06052	0.017	7	20
RPL10	0.06125	0.0174	7	Essential gene
KPNB1	0.06199	0.0177	7	21
ATP1A1	0.06937	0.021	7	22
EIF3G	0.07085	0.0213	5	Essential gene
NAE1	0.07232	0.0222	6	23
CDK9	0.0738	0.0229	7	24
TXN	0.0797	0.0253	5	25
RAD51	0.08044	0.0255	4	26
CLTC	0.08118	0.0261	6	27
HSP90B1	0.08192	0.0271	5	28
PSMB2	0.08339	0.0281	6	29
BIRC5	0.08413	0.0287	6	30
MDM2	0.09373	0.0337	5	31
POLA2	0.0952	0.0348	4	32
CDK1	0.1004	0.0381	6	33
KRAS	0.1048	0.0412	7	34
BRD4	0.107	0.0429	4	35

KIF11	0.1077	0.0434	6	36
BCL2L1	0.1122	0.046	7	37
AURKB	0.1137	0.0472	4	38
TP53	2.29	1	0	
CHEK2	2.08	1	0	
NOTCH2	1.97	1	0	

Supplementary Table 9: Comparison of shRNA and CRISPR hits for A549

Gene	shRNA Gene Rank	CRISPR Gene Rank
RPS6	Essential gene	Essential gene
VCP	1	11
PSMB5	2	15
SFPQ	Essential gene	Essential gene
EIF3G	Essential gene	Essential gene
MTOR	3	17
PSMD1	4	
PSMD2	5	
CDK1	6	33
AURKB	7	38
EWSR1	8	
UBA1	9	1
HSP90B1	10	28
XPO1	11	20
KIF11	12	36
BRD4	13	35
POLE	14	
NEDD8	15	14
CSNK1A1	16	
CLTC	17	27
TACC3	18	
KPNB1	19	21
CA6	20	
RPTOR	21	16
RPL7	Essential gene	Essential gene
DDB1	22	8
PSMB1	23	
POLA1	24	
SPTLC2	25	
USP7	26	10
ERBB2	27	
RRM1	28	
RAD51	29	26
RAN	30	4
PTK2	31	
TTK	32	
PI4KB	33	
VDR	34	

BCL2L1	35	37
CUL1	36	13

Supplementary Table 10: Listing of Broad CTD2 440 compound plates

PROJ_CPD_NAME	STATUS	TARGET_NODES
Zebularine	probe	CDA;DNMT1
EPZ004777 analog	probe	DOT1L
importazole	probe	KPNB1
SR8278	probe	NR1D1
BXL-628;elocalcitol	clinical	VDR
ML204	probe	IDH1
docetaxel;Taxotere	FDA	TUBB1;BCL2
cyclophosphamide;Endoxan;Cytoxan;Neosar;Procytox;Revimmune	FDA	
nutlin-3	clinical	MDM2
NVP-231	probe	CERK
triptolide	clinical	
avicin D	probe	NR3C1
Bax channel blocker;BRD3547	probe	BAX
CCT036477;BRD7170	probe	
erastin	probe	VDAC1;VDAC2
curcubitacin I;JSI-124	probe	NFKB1
paclitaxel;taxol	FDA	TUBB1
hyperforin	clinical	TRPC6
brefeldin A	probe	ARF1
itraconazole;Sporanox	FDA	
dexamethasone;Decadron	FDA	NR3C1
teniposide;Vumon;VM-26	FDA	TOP2A;TOP2B
ML162;BRD5421	probe	
necrostatin-1	probe	RIPK1
SN-38;7-ethyl-10-hydroxycamptothecin	probe	TOP1
ciclosporin;cyclosporin	FDA	
TGX-221	probe	PIK3CB
bleomycin	FDA	
968	probe	GLS
fluvastatin;Lescol	FDA	HMGCR
PX-12;IV-2	clinical	TXN
PD318088	probe	MAP2K1;MAP2K2
Col-3	clinical	MMP1
YK-4-279	probe	ERG;ETV1;EWSR1;FLI1
fumonisin B1	probe	CERS1;CERS2;CERS3;CERS4;CERS5;CERS6
ifosfamide;Ifex	FDA	
salermide	probe	SIRT1;SIRT2
BCL-LZH-4	probe	BCL2L1
isoevodiamine;evodiamine	probe	
lovastatin acid;NP-001236;Mevacor;Altocor;Altoprev	FDA	HMGCR
MH2075	probe	IDH1
blebbistatin	probe	MYH1;MYH2
myriocin;antibiotic ISP-1;thermozymocidin	probe	SPTLC1;SPTLC2;SPTLC3

IPR-456	probe	PLAUR
NSC23766	probe	RAC1;TRIO;TIAM1
oligomycin A	probe	ATP5L2
ML258	probe	BCL2L10
fulvestrant;Faslodex	FDA	ESR1
CID-5951923	probe	KLF5
thalidomide	FDA	KDR;TNF
temozolomide;Methazolastone;Temodar;Temodal	FDA	
cyanoquinoline 11	clinical	MAP3K8;EGFR
RITA;NSC652287	probe	TP53;MDM2
tubastatin A	probe	HDAC6
BRD-K01121114	probe	
LRRK2-IN-1	probe	LRRK2
SP600125	probe	MAPK8;MAPK9;MAPK10
BRD-K01737880	probe	
ML210;TL-HRAS-61	probe	
NVP-BSK805	probe	JAK2
olaparib;AZD-2281;KU-0059436	clinical	PARP1;PARP2
panobinostat;THM-I-91;LBH-589	clinical	HDAC1;HDAC2;HDAC3;HDAC6;HDAC8
BRD-K02251932	GE-active	
parbendazole	probe	
QS-11	probe	ARFGAP1
SM-406;AT-406	probe	XIAP;BIRC2;BIRC3
SU11274	probe	MET
HC-067047	probe	TRPV4
azacitidine	FDA	DNMT1
foretinib;XL880;EXEL-2880;GSK1363089;GSK089	clinical	MET;KDR
Merck60;BRD6929	probe	HDAC1;HDAC2
BIBR-1532	probe	TERT
BRD-K04800985	GE-active	
BMS-270394	probe	RARG
BIO;6-bromoindirubin-3'-oxime;6BIO	probe	JAK1;JAK2;JAK3;GSK3B
ML030;triazolothiadiazine	probe	PDE4A;PDE4B;PDE4D
BRD9647	probe	
PDMP;1-phenyl-2-decanoylamino-3-morpholino-1-propanol	probe	UGCG
LE-135	probe	RARB
GSK1059615	clinical	MTOR;PIK3CA;PIK3CB;PIK3CD;PIK3CG
narciclasine	probe	RHOA
AM-580	probe	RARA;RARB;RARG
AA-COCF3;arachidonyl trifluoromethyl ketone	probe	PLA2G4A;PLA2G4B;PLA2G4C;PLA2G4D;FAAH
GSK525762A;I-BET	probe	BRD2;BRD3;BRD4
temsirolimus;CCI-779;Torisel	FDA	MTOR
SID-26681509;BRD7745	probe	CTSL1
linsitinib	clinical	IGF1R;IGF1
N-hexanoyl-D-sphingosine;C6-ceramide	probe	UGCG;MAPK1;PPP2CA

BRD-K09344309	probe	
GANT-61	probe	GLI1;SMO
KU-0060648	probe	PRKDC
BRD-K09587429	GE-active	
FGIN-1-27	probe	TSPO
PRL-3 inhibitor I;BRD7482	probe	PTP4A3
dabrafenib;dabrafenib mesylate;GSK2118436	clinical	BRAF
GW-405833;L-768,242	probe	CB2
BO2	probe	RAD51
BRD-K11533227	probe	HDAC1;HDAC2
PF-184	probe	IKBKB
carboplatin	FDA	
AT7867	probe	AKT1;AKT2;AKT3;RPS6KB2
BEZ235;NVP-BEZ235	clinical	MTOR;PIK3CA;PIK3CB;PIK3CD;PIK3CG
trametinib;GSK1120212;JTP-74057	clinical	MAP2K1;MAP2K2
TG-101348;SAR302503	clinical	JAK2
valdecoxib;Bextra	FDA	PTGS2
procarbazine;Matulane	FDA	
ciclopirox olamine;Loprox	FDA	RRM1
BMS-754807	probe	IGF1R
BMS-345541	probe	IKBKB
dinaciclib;SCH727965	clinical	CDK1;CDK2;CDK5;CDK9
BRD-K13999467	GE-active	
BRD-K14844214	GE-active	
R428	probe	AXL
gemcitabine	FDA	RRM1;TYMS;CMPK
NVP-ADW742	probe	IGF1R
PRIMA-1	probe	TP53
phloretin;dihydronaringenin;Phloretol;naringenin chalcone	probe	
obatoclax	clinical	BCL2;MCL1;BCL2L1
CHIR-99021;CT99021	probe	GSK3A;GSK3B
PLX-4720	probe	BRAF
regorafenib;BAY-73-4506;Stivarga	FDA	FLT1;KDR;FLT4;PDGFRB;KIT;RET;RAF1
sotrastaurin;AEB071	clinical	PRKCB
BRD-K17060750	GE-active	
bendamustine;bendamustine hydrochloride;Treakisym;Ribomustin;Levact;Treanda	FDA	
SCH-79797	probe	F2R
HLI-373;DPD	probe	MDM2
indisulam;E7070	clinical	CA1;CA2;CA3;CA4;CA7;CA9;CA12;CA13;CA14
belinostat;PXD-101	clinical	HDAC1;HDAC2;HDAC3;HDAC6;HDAC8
TL-32711	clinical	DIABLO;XIAP
MK-0752	clinical	NCSTN;PSEN1;PSENEN;APH1A
BRD-K19103580	GE-active	
gossypol	clinical	BAX;BCL2;PRKCI
prochlorperazine;prochlorperazine dimaleate;Compazine	FDA	DRD2

sitagliptin	FDA	DPP4
SGX-523	clinical	MET
saracatinib;AZD0530	clinical	SRC;ABL1
lapatinib;GW572016;GW2016;Tykerb;Tyverb	FDA	EGFR;ERBB2
NVP-LDE225;BRD6430	probe	PARP1;PARP2
MST-312	probe	TERT
R-406	clinical	SYK
SKI-II	probe	SPHK1
KW 2449	clinical	FLT3;ABL1;AURKA
RO4929097	clinical	NCSTN;PSEN1;PSENEN;APH1A
simvastatin;Zocor	FDA	HMGCR
STF-31	probe	SLC2A1
1S,3R-RSL-3	probe	
sorafenib;BAY-43-9006;Nexavar	FDA	BRAF;RAF1;KDR;FLT3;FLT4;PDGFRB;KIT
piperlongumine	probe	
SCH-529074	probe	TP53
FSC231	probe	PICK1
fluorouracil;Adrucil;5-fluorouracil;5-FU;Efudex	FDA	TYMS
KU-55933	probe	ATM
OSI-930	clinical	MET;KDR
CD-1530	probe	RARG
BRD-K26531177	probe	
PD 153035	probe	EGFR
cytochalasin B	probe	
BIX-01294	probe	EHMT2
BRD-K27224038	GE-active	
leptomycin B	probe	XPO1
lomeguatrib	clinical	MGMT
BRD-K27986637	probe	
TW-37	probe	BCL2;BCL2L1
AZD4547	clinical	FGFR1;FGFR2;FGFR3
brivanib;BMS-582664	clinical	KDR
napthofuran compound 5	probe	HNF4A
CD-437;AHPN	probe	RARG
BRD-K29086754	GE-active	
BRD-K29313308	probe	HDAC3
PIK-93	probe	PIK3CG;PI4KB
chlorambucil;Leukeran	FDA	
NSC30930;5-(benzyloxy)-1H-indole-2-carboxylic acid	probe	
axitinib;AG013736;Inlyta	FDA	KDR;FLT4
KX2-391;KX01	clinical	SRC
BRD-K30019337	GE-active	
compound 1452A;BRD4966	probe	CASP3;CASP6;CASP7
CDK9 inhibitor 14	probe	CDK9
VU0155056	probe	PLD1

peranib-2	probe	ASAH1;ASAH2;ASAH2B;ASAH2C;ACER1;ACER2;ACER3
VER-155008	probe	HSPA1A;HSPA1B;HSPA1L
AGK-2	probe	SIRT2
cytarabine;cytosine arabinoside;Cytosar-U;Depocyt;Ara-C	FDA	
BRD-K33199242	GE-active	
tivantinib;ARQ-197	clinical	MET
BRD-K33514849	probe	
isoliquiritigenin;GU 17	probe	SIRT1
clofarabine;Clolar	FDA	POLA1;POLA2;POLE;RRM1
BRD-K34099515	probe	
cimetidine;Tagamet	FDA	HRH2
BRD-K34222889	probe	
BRD-K34485477	GE-active	
BMS-536924	probe	IGF1R;INSR
dacarbazine;DTIC-Dome	FDA	
ouabain	probe	ATP1A1;ATP1A2;ATP1A3;ATP1A4;ATP1B1;ATP1B2;ATP1B3;ATP1B4
BRD6340	probe	MYC
darinaparsin	clinical	
niclosamide;Niclocide	FDA	STAT3
KU-60019	probe	ATM
GSK1210151A;I-BET151	probe	BRD2;BRD3;BRD4
StemRegenin 1;SR1	probe	AHR
BRD-K37390332	GE-active	
SB-525334	probe	TGFBR1
PF-3758309	probe	PAK4
etoposide	FDA	TOP2A
LY-2183240	probe	FAAH
NSC19630	probe	WRN
lenvatinib;E7080	FDA	KDR;FLT4
S3I-201;NSC74859	probe	STAT3
BRD-K41334119	GE-active	
BRD-K41597374	GE-active	
palmostatin B	probe	LYPLA1
MM_V_GSK_2d1	probe	EZH2
sunitinib;Sutent;SU11248	FDA	FLT1;KDR;FLT4;FLT3;PDGFRB;KIT;RET;CSF1R
myricetin	probe	STX1A;VAMP1;SNAP25
SNS-032;BMS-387032	clinical	CDK2;CDK7;CDK9
MLN-2480	clinical	ARAF;BRAF;RAF1
PF-4800567 hydrochloride	probe	CSNK1D;CSNK1E
WP1130	probe	USP9X;USP5;USP14;UCHL5
BRD-K44224150	GE-active	
ASN-05257430;T6909415;BRD1590	probe	PKM
pandacostat	probe	HDAC1;HDAC2;HDAC3;HDAC6;HDAC8
betulinic acid	probe	SP1

BRD-K45681478	probe	
NSC48300	probe	TASP1
IU1	probe	USP14
BMS-195614	probe	RARA;RARB;RARG
Ki8751	probe	KIT;KDR;PDGFRA;FGFR2
GMX-1778;CHS-828	clinical	NAMPT
Repligen 136	probe	HDAC3
SR1001	probe	RORA;RORC
BRD-K48334597	GE-active	
nintedanib;BIBF 1120;Vargatef	clinical	FLT1;KDR;FLT4;PDGFRA;PDGFRB;FGFR1;FGFR2;FGFR3
GSK-2636771	clinical	PIK3CB
BRD-K49290616	GE-active	
dasatinib;BMS-354825;Sprycel	FDA	ABL1;SRC;EPHA2;LCK;YES1;KIT;PDGFRB;STAT5B;ABL2;FYN
PRIMA-1-Met;APR-246	probe	TP53
abiraterone;Zytiga	FDA	CYP17A1
sildenafil;Viagra;Revatio	FDA	PDE5A
NVP-TAE684	probe	ALK
necrostatin-7	probe	
canertinib;CI-1033	clinical	EGFR;ERBB2
HBX-41108	probe	USP7
BRD-K50799972	GE-active	
BRD-K51490254	probe	HDAC6;HDAC8
cabozantinib;Cometriq;XL184;BMS-907351	FDA	MET;FLT3;KDR;KIT;TEK
TPCA-1	probe	NFKB1
IKK-3 inhibitor IX	probe	IKBKE
BRD-K52037352	GE-active	
cerulenin	probe	FASN;HMGCS1
CI-994	probe	HDAC1;HDAC2;HDAC3;HDAC6;HDAC8
methylstat	probe	KDM3A;KDM4A;KDM4B;KDM4C;KDM4D;JMJD6
AZD7545	probe	PDK2
GDC-0941	clinical	PIK3CA;PIK3CB;PIK3CD;PIK3CG
Chembridge cat# 7667791	probe	TNKS
tivozanib;AV-951;BRD4658	clinical	KDR
BEC;S-(2-boronoethyl)-L-cysteine	probe	ARG1;ARG2
SRT-1720	probe	SIRT1
BRD3639;CHEMBL1222381;neuronal differentiation inducer III,;NDI III	probe	NEUROD1;NEUROD2
ruxolitinib;INCB018424;Jakafi;Jakavi	FDA	JAK1;JAK2
MK-1775	clinical	WEE1
JQ-1	probe	BRDT
WAY-362450;FXR450	probe	NR1H4
BYL-719	clinical	PIK3CA
BRD-K55116708	probe	
SZ4TA2	probe	BCL2L1
rigosertib;ON-01910;Estybon;Novonex	clinical	PLK1
BRD-K55478147	probe	HDAC6

epigallocatechin-3-monogallate	probe	
topotecan;Hycamtin	FDA	TOP1
MGCD-265	clinical	FLT1;KDR;FLT4;MET;TEK
ABT-737	clinical	BCL2;BCL2L1;BCL2L2
vemurafenib;PLX-4032;RG7204;Zelboraf;RO5185426	FDA	BRAF
selumetinib;AZD6244	clinical	MAP2K1;MAP2K2
marinopyrrole A;maritoclax	probe	MCL1;ACTB
AT13387	clinical	HSP90AA1
APO866;FK866;BRD0667	clinical	NAMPT
AZD6482	clinical	PIK3CB
AZ-3146;Mps1-IN-2	probe	TTK
DBeQ	probe	VCP
tozasertib;VX-680;MK-0457	clinical	AURKA;AURKB;AURKC
bardoxolone methyl;CDDO-methyl ester	clinical	
methotrexate;Abitrexate	FDA	DHFR
Mitomycin C	FDA	
BRD2020;CHEMBL374350	probe	NFKB1
JNJ-26854165;Serdemetan	clinical	MDM2
PYR-41	probe	UBA1
CAL-101	clinical	PIK3CD
Mdivi-1	probe	DNM1
BRD-K61166597	probe	HDAC1;HDAC2
SB-225002	probe	CXCR2
CAY10594;compound 72	probe	PLD2
compound 7d-cis;BRD9047	probe	XPO1
SB-743921	clinical	KIF11
ABT-199	clinical	BCL2
HMN-214	probe	PLK1
tipifarnib-P2	probe	FNTA
ZSTK474	clinical	PIK3CG
tipifarnib-P1;Zarnestra	clinical	FNTA
BRD-K63431240	probe	
barasertib;AZD1152-HQPA	clinical	AURKB
gefitinib;ZD-1839;Iressa	FDA	AKT1;EGFR
necrosulfonamide	probe	
MI-1	probe	MEN1;MLL
apicidin	probe	HDAC1;HDAC2;HDAC3;HDAC6;HDAC8
ATRA;all-trans retinoic acid;tretinoin;Vesanoid	FDA	RARA;RARB;RARG
Ko-143	probe	ABCG2
TG100-115	clinical	PIK3CD;PIK3CG
PHA-793887	clinical	CDK1;CDK2;CDK4;CDK5;CDK7;CDK9
BI-2536	clinical	PLK1
semagacestat;LY450139	clinical	NCSTN;PSEN1;PSENEN;APH1A
AZD1480	clinical	JAK1;JAK2
afatinib;BIBW2992	clinical	EGFR;ERBB2
KH-CB19	probe	CLK1;CLK4

BRD-K66453893	GE-active	
pifithrin	probe	
SB-431542	probe	TGFBR1
KU-0063794	probe	MTOR
GDC-0879	probe	BRAF
MLN-4924	clinical	NAE1
PI-103	probe	MTOR;PIK3CA;PIK3CB;PIK3CD;PIK3CG;PRKDC
MK-2206	clinical	AKT1;AKT2;AKT3
NSC95397	probe	CDC25A;CDC25B;CDC25C
BRD8958;C646;OSSK-701387	probe	EP300
alvocidib;flavopiridol	clinical	CDK1;CDK2;CDK4;CDK6
cisplatin;CDDP;Platin	FDA	
tacrolimus;FK-506;fujimycin;Prograf;Advagraf;Protopic	FDA	PPP3CA;PPP3CB;PPP3CC;PPP3R1;PPP3R2
ISOX;BRD-K69840642	probe	HDAC6
AZD8055	clinical	MTOR
ibrutinib;PCI-32765	clinical	BTK
erlotinib;CP-358774;OSI-774;NSC718781;Tarceva	FDA	EGFR;ERBB2
BRD-K70809902	probe	SCARB1
masitinib;Masivet	clinical	KIT;PDGFRA;PDGFRB;FGFR3
SNX-2112	probe	HSP90AA1
Ch-55	probe	RARA;RARB;RARG
N9-isopropylolomoucine	probe	CDK1;CDK5
6630-29-1	probe	
QW-BI-011;BRD4770	probe	EHMT2;EZH2
WZ4002	probe	EGFR
BRD1812	probe	CREB1;CTNNB1;CREBBP
purmorphamine	probe	SMO
BIRB-796;Doramapimod	probe	MAPK14
curcumin	clinical	PPID
UNC0321	probe	EHMT2
NSC632839	probe	USP5;USP13
pazopanib;pazopanib hydrochloride;GW786034;Votrient;Armala	FDA	KDR;FLT1;FLT4;KIT;PDGFRB
BRD-K75293299	GE-active	
alisertib;MLN8237	clinical	AURKA
XL765;SAR245409	clinical	MTOR;PIK3CA;PIK3CB;PIK3CD;PIK3CG
IC-87114	probe	PIK3CD
JW74	probe	CTNNB1;GSK3B;AXIN1;APC;CSKN1A1
omacetaxine mepesuccinate;homoharringtonine;Synribo	FDA	
YM-155	clinical	BIRC5
A-804598	probe	P2RX7
EX-527;SEN0014196	clinical	SIRT1
vandetanib;Zactima;ZD6474;Caprelsa	FDA	KDR
entinostat	clinical	HDAC1;HDAC2;HDAC3;HDAC6;HDAC8
LY-2157299	probe	TGFBR1
crizotinib;PF-2341066;Xalkori	FDA	ALK;MET

BRD-K78574327	GE-active	
manumycin A	probe	FNTA;FNTB
MLN2238;MLN9708	clinical	PSMB5
GSK4112	probe	NR1D1
KHS101	probe	TACC3
PF-573228	probe	PTK2
decitabine	FDA	DNMT1
PF-543	probe	SPHK1
ML203;NCGC00185916-03;compound 58;ms-ctdd-3;BRD2993	probe	PKM
etimoxir	clinical	CPT1A
vorinostat;SAHA;Zolinza	FDA	HDAC1;HDAC2;HDAC3;HDAC6;HDAC8
silmitasertib;CX-4945	clinical	CSNK2A1;CSNK2A2
tanespymicin;17-AAG	clinical	HSP90AA1
BRD-K81491172	probe	GSK3B
nilotinib;AMN107;Tasigna	FDA	BCR;ABL1;KIT
parthenolide	probe	
WZ8040	probe	EGFR
vincristine;Oncovin;leurocristine	FDA	TUBB1
navitoclax	clinical	BCL2;BCL2L1;BCL2L2
PF-750	probe	FAAH
CAY10618	probe	NAMPT
PL-DI;piperlongumine-dimer	probe	
skepinone-L	probe	MAPK14
nelarabine	FDA	POLA1
CBB-1007	probe	KDM1A
BRD-K84807411	GE-active	
sirolimus;rapamycin;Rapamune	FDA	MTOR
spautin-1	probe	USP10;USP13
BRD-K85133207	probe	HDAC1
neratinib;HKI-272	clinical	EGFR;ERBB2
AZD7762	clinical	CHEK1;CHEK2
BRD-K86535717	GE-active	
BRD4132	probe	MYC
bafilomycin	probe	ATP6V0A1
UNC0638	probe	EHMT1;EHMT2
cediranib;AZD2171	clinical	FLT1;KDR;FLT4
OSI-027	clinical	MTOR;RPTOR;MLST8;RICTOR;MAPKAP1
veliparib;ABT-888	clinical	PARP1;PARP2
L-685458	probe	NCSTN;PSEN1;PSENEN;APH1A
CYT-387	clinical	JAK1;JAK2
istradefylline;KW-6002	clinical	ADORA2A
fingolimod;FTY720;Gilenya	FDA	S1PR1
bortezomib	FDA	PSMD1;PSMD2;PSMB1;PSMB2;PSMB5
CI-976	probe	ACAT1
BRD-K88742110	probe	HDAC8

tandutinib;MLN518;CT53518	clinical	FLT3;KIT;PDGFRB
BRD9876	probe	WEE1
RG-108	probe	DNMT1
ML006;T6748133;BRD2698	probe	S1PR3
trifluoperazine;Stelazine	FDA	DRD2
GW-843682X	probe	PLK1;PLK3
pitstop2	probe	CLTA;CLTB;CLTC;CLTCL1
bosutinib;SKI-606;Bosulif	FDA	SRC;ABL1
MI-2	probe	MEN1;MLL
O-6-benzylguanine	clinical	AGXT
doxorubicin;adriamycin;hydroxydaunorubicin	FDA	TOP2A
tosedostat;CHR-2797	clinical	LAP3;NPEPPS;ANPEP
GSK461364	clinical	PLK1
bexarotene;Targretin	FDA	RXRA;RXRB;RXRG
imatinib;STI571;Gleevec	FDA	BCR;ABL1
BRD-K92856060	probe	
SMER-3	probe	SKP1;CUL1
PAC-1	probe	CASP3
SJ-172550	probe	TP53;MDM2
RAF265;CHIR-265	clinical	BRAF;KDR
AC55649	probe	RARB
BRD-K98948170	probe	
vorapaxar;SCHá530348	clinical	F2R
tamoxifen;Nolvadex;Istubal;Valodex	FDA	ESR1;ESR2
quizartinib;AC220	clinical	FLT3
CHM-1	probe	
VAF-347	probe	AHR
BRD-K96431673	GE-active	
pifithrin-mu;NSC303580	probe	TP53;HSPA1A;HSPA1B;HSPA1L
BRD-K96970199	GE-active	
GSK-J4	probe	KDM6A;KDM6B
16-beta-bromoandrosterone	probe	PTGER3;PTGER4
pluripotin	probe	MAPK3;RASA1
JW-480	probe	NCEH1
linifanib;ABT-869	clinical	KDR;FLT1;FLT3;CSF1R;PDGFRB
Infinity compound 1	probe	BCL2
oxaliplatin;Eloxatin	FDA	

Supplementary Table 11: AUCs <0.5 of CLF-PED-015-T to Broad CTD2 440 compound plates

Compound	CLF-PED-015-T passage 13	Target
daporinad	0.13409	NAMPT
avicin D	0.16323	AMPK
narciclasine	0.18794	GTPase alkaloid
leptomycin B	0.21489	XPO1
bortezomib	0.22442	Proteosome inhibitor
GMX-1778	0.23613	NAMPT
PL-DI	0.26869	ROS
MST-312	0.28937	TERT
brefeldin A	0.29439	Anterograde transport
YM-155	0.30169	BIRC5
SN-38	0.30502	TOP1
LBH-589	0.3064	HDAC
triptolide	0.31202	Unknown
omacetaxine mepesuccinate	0.33426	Protein translation
dinaciclib	0.34462	CDK inhibitor
ouabain	0.3555	Na/K atpase
alvocidib	0.36712	CDK inhibitor
BI-2536	0.39749	PLK
SMER-3	0.41192	SCFMET30
PF-3758309	0.41405	PAK
doxorubicin	0.41495	TOP2A
vincristine	0.42718	Inhibitor of microtubule assembly
topotecan	0.43789	TOP1
KX2-391	0.44497	SRC inhibitor
SNS-032	0.46309	CDK inhibitor
parbendazole	0.46443	Inhibitor of microtubule assembly
BRD-K30748066	0.49157	Unknown
cucurbitacin I	0.49487	STAT3/JAK2
CAY10618	0.49708	NAMPT
AT13387	0.49832	HSP90
bardoxolone methyl	0.49887	IKK

Supplementary Table 12: Primers for fusion validation by qRT-PCR

Fusion Name	Primer pair (5'-3')
RPS24-EMP1	TTGGTGGTGGCAAGACAACT
	GGCTGTCACTGCAGCTAATG
VEGFA-DTNB	TCACCAAGGCCAGCACATAG
	CCATGGGTGGGCGATGTATG
UBXN7-GRID2	AGCTGATGTTGCGGTATCCA
	AGGGCCAAGATGCCTTGATT
IGF2BP2-AMFR	AGGTAAAGTGGAATTGCATGGG
	CTCACTCACTCGAAGAGGGC
FUS-GABRA3	TGCTCAGAGCGGAGAAAGC
	GCGATGTATGTGGTGACCCT
FSD1-CLSTN2	GCATCCGGCAGACAGAGTACA
	GCCCTCTCCTGATGTCTTGTT
GALNT7-PDLIM1	GTGGTCCTCTGGTCTTCCCTG
	TTCTTTGTCGATGACAAGGCTG
PRKCH-ALPK2	GGGTAAATGCGGTGGAACTTG
	CCCCAAAGTAGCTGTCATGGAG
ZNF680-XPA	GCCCTGGAATAGGAAGAGACAG
	CCCTTTTCCACACGCTGCTT
TMED10-ITPR2	AGGAGATTCACAAGGACCTGCTA
	TGAACAGTTCTCCTTGGCACAT
GAPDH	GAAGGTGAAGGTCGGAGTC
	GAAGATGGTGATGGGATTTC
TBP	TTCGGAGAGTTCTGGGATTGTA
	TGGACTGTTCTTCACTCTTGGC

Supplementary Table 13: shRNA/sgRNA constructs used for validation

shRNA

Clone ID	Target Gene	Gene Symbol	Taxon	Target Region	Target Seq
TRCN0000297275	1019	CDK4	human	3UTR	CCTTCCCATTTCTCTACACTA
TRCN0000279887	1019	CDK4	human	CDS	GTGGAGTGTTGGCTGTATCTT
TRCN0000279953	1019	CDK4	human	CDS	ACAGTTCGTGAGGTGGCTTTA
TRCN0000279952	1019	CDK4	human	3UTR	GAGATTACTTTGCTGCCTTAA
TRCN0000338462	7514	XPO1	human	CDS	GCTCAAGAAGTACTGACACAT
TRCN0000338401	7514	XPO1	human	CDS	ATTCGACTTGCGTACTCAAAT
TRCN0000338399	7514	XPO1	human	CDS	CCTCACCTACAAGATGCTCAA
TRCN0000072213	-12	RFP	CONTROL	CDS	ACTACACCATCGTGGAACAGT
TRCN0000231725	-12	RFP	CONTROL	CDS	ACTACACCATCGTGGAACAGT
TRCN0000072254	-14	LUCIFERASE	CONTROL	CDS	ATGTTTACTACACTCGGATAT
TRCN0000231737	-14	LUCIFERASE	CONTROL	CDS	ATGTTTACTACACTCGGATAT
TRCN0000072225	-15	lacZ	CONTROL	CDS	CTCTGGCTAACGGTACGCGTA

Clone ID	Target Gene	Known seed?	shRNA label	Gene
**TRCN0000279953**	**1019**	**Seed pair 1* no seed effect**	**CDK4-1**	**CDK4**
TRCN0000279952	1019	Seed pair 4	CDK4-2	CDK4
TRCN0000279887	1019	Seed pair 6	CDK4-3	CDK4
**TRCN0000297275**	**1019**	**Not in list**	**CDK4-4**	**CDK4**
**TRCN0000338401**	**7514**	**Seed pair 2* no seed effect**	**XPO1-1**	**XPO1**
TRCN0000338462	7514	Seed pair 4	XPO1-3	XPO1
**TRCN0000338399**	**7514**	**Not in list**	**XPO1-4**	**XPO1**

sgRNA

Gene Symbol	Taxon	Target Seq
TP53 (clone 3)	human	CACCGCCCCTTGCCGTCCCAAGCAA
GFP (clone 3)		AGCTGGACGGCGACGTAAAG
